# Is there adaptation in the human genome for taste perception and phase I biotransformation?

**DOI:** 10.1186/s12862-019-1366-7

**Published:** 2019-01-31

**Authors:** Begoña Dobon, Carla Rossell, Sandra Walsh, Jaume Bertranpetit

**Affiliations:** 1 Institut de Biologia Evolutiva (UPF-CSIC), Universitat Pompeu Fabra, Dr. Aiguader, 88. 08003 Barcelona, Catalonia Spain; 2 Department of Medical Biochemistry and Biophysics, Karolinska Institutet, Tomtebodavägen 23a, 17165 Stockholm, Solna Sweden

**Keywords:** Taste perception, Biotransformation, Cytochrome P450, Natural selection, *CYP3A4*, *CYP3A43*, *CYP27A1*

## Abstract

**Background:**

During the modern human expansion, new environmental pressures may have driven adaptation, especially in genes related to the perception of ingested substances and their detoxification. Consequently, positive (adaptive) selection may have occurred in genes related to taste, and in those related to the CYP450 system due to its role in biotransformation of potentially toxic compounds. A total of 91 genes (taste receptors and CYP450 superfamily) have been studied using Hierarchical Boosting, a powerful combination of different selection tests, to detect signatures of recent positive selection in three continental human populations: Northern Europeans (CEU), East Asians (CHB) and Africans (YRI). Analyses have been refined with selection analyses of the 26 populations of 1000 Genomes Project Phase 3.

**Results:**

Genes related to taste perception have not been positively selected in the three continental human populations. This finding suggests that, contrary to results of previous studies, different allele frequencies among populations in genes such as *TAS2R38* and *TAS2R16* are not due to positive selection but to genetic drift. *CYP1* and *CYP2* genes, also previously considered to be under positive selection, did not show signatures of selective sweeps. However, three genes belonging to the CYP450 system have been identified by the Hierarchical Boosting as positively selected: *CYP3A4* and *CYP3A43* in CEU, and *CYP27A1* in CHB.

**Conclusions:**

No main adaptive differences are found in known taste receptor genes among the three continental human populations studied. However, there are important genetic adaptations in the cytochrome P450 system related to the Out of Africa expansion of modern humans. We confirmed that *CYP3A4* and *CYP3A43* are under selection in CEU, and we report for the first time *CYP27A1* to be under positive selection in CHB.

**Electronic supplementary material:**

The online version of this article (10.1186/s12862-019-1366-7) contains supplementary material, which is available to authorized users.

## Background

There are two physiological processes related to the selection and ingestion of food that are important for the survival in humans: taste perception and phase I biotransformation. While taste perception is essential for food preferences and aversion, biotransformation can neutralize potential adverse effects that xenobiotics may have in an individual’s body. As modern humans expanded across continents and faced different environmental pressures, it is likely that natural selection would have operated on genes participating on taste perception and phase I biotransformation.

Taste perception is a key element in the interaction between organisms and the environment, as it provides crucial information about the quality and nutritional value of the food consumed and allows humans to detect potential dietary hazards. Humans can perceive five main different tastes: sweet, bitter, sour, salty and umami (the taste of L- glutamate) [1]. Perception of each of these tastes offers unique and essential information about the physiology and toxicity of different compounds. Sweet taste indicates the presence of carbohydrates, which serve as an energy source. Umami taste represents a food’s protein content. Sour perception detects ingestion of acidic substances (typically present in spoiled foods) and it causes food rejection. The same happens with bitter taste – it is also innately aversive – and protects organisms against toxins, which mostly taste bitter [2, 3]. Salty taste regulates the intake of sodium salts that are essential for the homeostasis of the body [4].

The other key process is biotransformation: the conversion of certain lipophilic substances into more polar substances easier to excrete; it affects many xenobiotics, such us alkaloids, pyrolysis products from cooked foods, secondary metabolites or toxins that can be found in plants, animals or fungi which are easy to absorb but difficult to eliminate. In the process of biotransformation, the essential part is phase I, carried out by cytochrome P450 (CYP) enzymes [5] and is based on oxidation, reductive and hydrolytic reactions that convert the original substance into a more polar one. Cytochrome P450 is a superfamily of highly polymorphic enzymes that comprises 57 genes that present allele frequency differences in human populations. These polymorphisms can affect the rate at which the enzyme’s substrates are transformed [6]. Selection may have acted upon these genes, possibly because each population was exposed to diverse sources of food and xenobiotics, thus forcing individuals to adapt. It has been hypothesized that a strong genetic differentiation among human groups may exist related to taste perception and xenobiotic defenses [1, 5, 6].

### The genetics of taste perception

Taste perception starts in the taste papillae found in epithelial surfaces of the tongue and the palate. Each papillae contains one or more taste buds, which in turn are formed by up to 100 cells containing specialized taste receptors [4]. These cells detect tastants and transduce the stimuli to afferent neurons relaying signals to brain structures involved in central processing of taste [7]. There are two main families of taste receptors mediating the perception of umami, sweet and bitter tastes: *TAS1R* and *TAS2R*, which are G-coupled chemoreceptors (GPCRs). Sour and salty taste are perceived through ion channels [1].

In humans, the *TAS1R* family consists of 3 genes: *TAS1R1, TAS1R2* and *TAS1R3* [8]. The proteins encoded by these genes form different heterodimers, each determining the perception of a specific taste. While the heterodimer *TAS1R2/TAS1R3* acts as a sweet taste receptor, the *TAS1R1/TAS1R3* perceives umami taste [1, 9]. The study of different evolutionary genetic parameters drove to the conclusion that variation in the *TAS1R* gene family has been under positive natural selection [10, 11].

The *TAS2R* gene receptor family has 25 functional genes that encode for bitter taste receptors in humans [12]. It is the most studied family among taste receptors, with most functional and evolutionary studies focusing on how the natural genetic variation in *TAS2R16* and *TAS2R38* influence bitter taste perception in humans. *TAS2R38* mediates the ability to taste phenylthiocarbamide (PTC) [13, 14] and 6-n-propylthiouracil [14]. Even though PTC is not directly found in food, its taste is associated with the identification of bitter compounds. Specific haplotypes at the *TAS2R38* locus have been associated with different taster phenotypes: the taster phenotype (PAV) with increased PTC sensitivity, the non-taster phenotype (AVI) with PTC insensitivity and intermediate sensitivities to PTC (AAV, AAI, and PVI) [13, 15]. The ability to taste PTC varies widely between individuals and among populations: only 35% of the Orissa population in India is a “taster”, while 90% of African Americans are tasters [16]. Different hypotheses have been proposed to explain these differences: recent positive selection [17], balancing selection maintaining both taster and non-taster alleles [18], or demographic events, such as a series of bottlenecks and population expansions with a relaxation of the selective forces [16]. *TAS2R16*, mediates the perception of salicin, amygdalin, and other bitter β-glucopyranosides, which are compounds that exist in nature and many have a highly toxic cyanogenic activity [19]. Differences in the sensitivity to cyanogenic glycosides in human populations have been attributed to positive selection towards a high salicin sensitivity [20, 21] or heterozygous advantage maintained by balancing selection [11]. Although these results suggest that natural selection acted upon some genes of the *TAS2R* family, a study proposed that *TAS2R* genes have been mostly under neutral evolution in humans [22]. In this study, they found neither *TAS2R16* nor *TAS2R38* under selection, and suggested that the finding of *TAS2R19* and *TAS2R45* being positively selected could be a false positive. It is important to note that the list of genes involved in taste cannot be considered complete and that for all these genes, beyond their involvement in taste, other functions have been described for each of them [23, 24].

Ion channels are at the base of sour and salty tastes. *PKD2L1*-expressing taste receptor cells have been shown to mediate the acid response in mice [25]. Although some studies have described the potential role of *PKD1L3* as another candidate for sour detection, targeted deletion of *PKD1L3* gene did not affect action potential firing after acid exposure [26–28]. Other recent candidates such as the proton selective channel *OTOP1* are also expressed in *PKD2L1*-expressing taste receptor cells and were shown to mediate acid responses as well [29]. Additional functional studies are needed to understand the exact mechanism of sour taste responses. As for salty taste, studies suggest that its perception involves a selective ion channel named epithelial amiloride-sensitive sodium channel (*ENaC*) [30, 31]. This ion channel has four subunits, α, β, γ, and δ that are encoded by the genes *SCNN1A, SCNN1B, SCNN1G,* and *SCNN1D* respectively [32, 33]. A functional ENaC receptor consists of three subunits αβγ or δβγ [34], but most evidence indicates that αβγ is the main actor involved in salty taste transduction in taste receptor cells [35]. It is known that these channels also play other roles in the body and are broadly expressed in different tissues [34–37]. To our knowledge, this is the first study that tries to unravel natural selection in either sour or salty receptors.

### Cytochrome P450

The cytochrome P450 superfamily consists of 57 genes in humans, which are classified into 18 families [38] (Table 1). Biotransformation is carried out by the *CYP1, CYP2, CYP3* families and in a much lesser degree, by the *CYP4* family [38, 39].

**Table 1:** Cytochrome P450 families with the number of genes and their main function

Gene family	Number of genes	Main function
CYP1	3	Biotransformation
CYP2	16	Biotransformation
CYP3	4	Biotransformation
CYP4	12	Fatty acids metabolism
CYP5	1	Thromboxane A2 synthesis
CYP7	2	Bile acid biosynthesis
CYP8	2	Bile acid and prostacyclin biosynthesis
CYP11	3	Steroid biosynthesis
CYP17	1	Steroid biosynthesis
CYP19	1	Steroid biosynthesis
CYP20	1	Unknown
CYP21	1	Steroid biosynthesis
CYP24	1	Vitamin D degradation
CYP26	3	Retinoic acid hydroxylation
CYP27	3	Vitamin D3 and bile acid synthesis
CYP39	1	Cholesterol synthesis
CYP46	1	Cholesterol synthesis
CYP51	1	Cholesterol synthesis

*CYP1* gene family is comprised of 3 enzymes: *CYP1A1*, *CYP1A2*, *CYP1B1*. They are activated by the aryl hydrocarbon receptor [40]. These enzymes metabolize polycyclic aromatic hydrocarbons, arylamines, and N-heterocyclics that are found in grilled food or meat cooked at high temperatures. These molecules can be carcinogenic and toxic when accumulated in the organism [41, 42]. Three independent studies found evidence of positive selection in *CYP1A2* gene. The first one was a resequencing study of *CYP1A2* gene where four derived SNPs were found at high allelic frequencies in human populations, indicating a possible event of positive selection [43]. Two other studies found evidence of a selective sweep in *CYP1A2* by using haplotype-based methods in worldwide populations with discrepant results: Li et al. [44] reported *CYP1A2* and *CYP1A1* under positive selection only in African populations from the International HapMap Project Phase III and the Human Genome Diversity Panel (CEPA-HGDP); whereas in another study *CYP1A2* was found under positive selection only in Europeans from HapMap Phase 1 [45].

*CYP2* is the largest *CYP* family, with 16 enzyme-coding genes. The most important genes in this family are *CYP2D6* and *CYP2C19* for their clinical relevance, as they are able to metabolize more than 100 drugs, clearly considered xenobiotics [39]. For these two genes the relationship between phenotype and genotype is well-established. Therefore, depending on the allele variant, an individual can be classified as: poor metabolizer (PM), meaning that the allele is null and therefore there is a lack of function of the enzyme; extensive metabolizer (EM), which would be the normal and most prevalent phenotype; intermediate metabolizer (IM) meaning that the person carries both a normal allele and a null one therefore the function is impaired; and finally, the ultra-rapid metabolizer (UM) in which the individual presents a gain-of-function variant. Both slow and ultra-rapid metabolizers are very rare phenotypes. This is relevant when evaluating hypotheses about whether these genes could be positively selected, as having gain of function variants results in higher clearance and lower xenobiotic concentrations. Loss of function variants, in turn, mean lower clearance rate and therefore higher xenobiotic concentrations. Janha et al. used linkage disequilibrium tests to detect positive selection and argued that some of the *CYP2C19* polymorphisms could have been positively selected in non-African populations and, when comparing African, Asian and European populations, the authors found that the slow metabolizing alleles had been positively selected within the last 10,000 years [46]. Other *CYP2* genes have been detected under positive selection in several populations: *CYP2C8*, *CYP2C9*, *CYP2D6* and *CYP2E1* have been positively selected worldwide, while *CYP2A6* and *CYP2B6* were found under positive selection only in African populations [44]. Voight et al. found positive selection for *CYP2E1* only in Europeans [45]*.*

The other gene family participating in biotransformation is the *CYP3*, composed of the enzymes *CYP3A4*, *CYP3A5*, *CYP3A7* and *CYP3A43*. These enzymes are all located in a cluster known as the *CYP3A* locus. They are particularly relevant in humans as they are involved in the biotransformation of a wide variety of compounds, such as drugs and many naturally occurring flavonoids, pyrrolizidine alkaloids, herbal constituents, and food derived retinoids. *CYP3A4* is the most abundantly expressed *CYP* in the liver and small intestine and has a crucial role in drug metabolism as it metabolizes more than 50% of the drugs available in market [47–50]. Previous studies have reported signatures of positive selection in the *CYP3A* locus by using neutrality tests based on site-frequency spectrum and haplotype structure on African, European and Asian populations. Specifically, Chen et al. [51] found that *CYP3A4* and *CYP3A7* have been under positive selection in Europeans, East Asians and Africans, while *CYP3A5* was found under positive selection only in non-Africans populations. Another study found that *CYP3A4* and *CYP3A5* had been under selection in a set of worldwide populations, but no evidence of positive selection was found in *CYP3A7* [44]. Voight et al. found evidence of positive selection in *CYP3A5* only in *Europeans* [45]*.*

The *CYP4* gene family is the largest after the *CYP2*. It also participates in biotransformation and drug metabolism, but its main function is related to fatty acid metabolism. Most of the genes in this family are considered “orphan” as their function is still unclear [39, 52], including a cluster of *CYP4* genes (*CYP4B1, CYP4A11, CYP4X1, CYP4Z1, CYP4A22*) found under positive selection in Europeans [45].

The rest of CYP families consist on one to three genes and their main function is the biosynthesis of endogenous compounds [38, 39]. Among them, only *CYP21A2* has been described to be under selection, although we think that more evidence is needed in order to confirm this results [53]. *CYP21A2* participates in the production of steroid hormones and it is located within the HLA locus [39].

The objective of this study is to find whether known taste receptor genes and cytochrome P450 genes have been positively selected in humans by analyzing the data of the 1000 Genomes Project Phase 1 and Phase 3. To our knowledge, this is the first work to study all known genes related to these functions, including the ion channels, with a robust and uniform analysis to detect footprints of past events of positive selection.

## Results

We inspected signatures of positive selection in genes related to taste perception and phase I biotransformation in human populations from the 1000 Genomes Project (Phase 1 and Phase 3). To identify selective sweeps, we used the Hierarchical Boosting (HB) [54], a machine learning classification method that integrates the information from several selection tests to detect selective sweeps under the hard sweep model. The advantage of using a composite method is that they have higher power than single tests and a lower rate of false positive signals [55–57]. Moreover, the HB also accounts for population-specific demography, a confounding factor in selection studies [58]. HB has been able to validate well-known loci under positive selection in European populations, such as *SLC24A5* [59, 60] (Additional file 1: Figure S1a) and *LCT* [59, 61] (Additional file 1: Figure S1b); *EDAR* in Asian populations [55, 59] (Additional file 1: Figure S1c) or *DARC* in African populations [62] (Additional file 1: Figure S1d), and among others, has been used to detect positive selection in micronutrient and protein quantitative trait loci [63], and in common risk alleles associated to autism spectrum disorder [64]. We also identified signals of positive selection with single statistics based on population differentiation, site frequency spectrum and linkage disequilibrium structure (Additional file 2: Table S2) from the 1000 Genomes Selection Browser 1.0 for Phase 1 [65] and from the PopHuman Browser for Phase 3 [66].

### No evidence of hard selective sweeps in taste receptors genes

When we investigated the *TAS1R* receptor family, we did not detect signals of selective sweeps in *TAS1R1* or *TAS1R2* in the HB. Furthermore, none of the individual selection tests (F_ST_, deltaDAF, iHS, Tajima’s D, XP-CLR or XP-EHH) gave any indication of positive selection in those genes. For *TAS1R3*, there was no information in the HB and among the individual selection tests, only two SNPs in the promoter region of *TAS1R3* exhibited significant deltaDAF scores when comparing CEU (European) with CHB (Asian) and YRI (African) populations (Additional file 1: Figure S2), making the action of positive selection in this locus very unlikely.

Focusing on the *TAS2R* family, none of the genes showed signals of a hard sweep; including those previously reported under positive selection [17, 20, 22] (Additional file 2: Table S1). We also checked if balancing selection had acted on *TAS2R38* and *TAS2R16* as some studies have reported [11, 18], but none of the populations have a significant value of Tajima’s D (with a rank score > 2 or above 2 standard deviations (sd) of the mean), suggesting that balancing selection is not at the base of the variation on these genes (Additional file 1: Figure S3).

Regarding the ion channels, the HB showed no signals of positive selection either in the genes related to sour tasting (*PKD2L1, PKD1L3 or OTOP1*) (Additional file 1: Figure S4) or in the genes related to salty taste perception (*SCNN1A*, *SCNN1B* and *SCNN1G*) (Additional file 1: Figure S5a-b). No conclusion can be made on *SCNN1D* since there is no HB or haplotype-based information (XP-EHH and iHS) in PopHuman Phase 3. A few SNPs show significant scores when comparing CEU with CHB or YRI in deltaDAF and in XP-CLR when comparing CEU with CHB or YRI (Additional file 1: Figure S5c).

Thus, as a general result, no signals of positive or balancing selection have been detected in any human population for the known genes at the base of taste perception.

### Cytochrome P450 genes

#### No positive selection on CYP1, CYP2 and CYP4 genes

None of *CYP1*, *CYP2* and *CYP4* genes previously described under positive selection [43–46] show a signature of a selective sweep in the HB; even though some of them have high values in individual tests that may have been previously interpreted as selective events. For example, *CYP1A2* does not show any signature of positive selection in the HB in YRI (Additional file 1: Figure S6), contrary to previous results [44]. *CYP2E1* has high XP-EHH and F_ST_ values (above 2 sd of the genome-wide mean) in the comparison between CEU and YRI (Additional file 1: Figure S7a). Values of iHS for most European and African populations (including CEU and YRI) are also above 2 sd of the genome-wide mean. Nonetheless, the HB does not reach significance in CEU or YRI, suggesting a false positive in the studies that used single selection tests [54] (Additional file 1: Figure S7b).

In the case of *CYP4* genes, *CYP4B1* and *CYP4Z1* are located in the same cluster and were previously detected to be under positive selection in Europeans using an haplotype-based method (iHS) [45]. *CYP4Z1* shows significant values of iHS in most Europeans and Indian populations from Phase 3 and F_ST_ values above 2 sd when comparing CHB with CEU or YRI near the promoter region. *CYP4B1* has significant iHS values in European, Asian and Indian populations (Additional file 1: Figure S8a). However, HB does not give a significant result in any of the genes in the cluster (Additional file 1: Figure S8b).

*CYP4F3* and *CYP4F12* have no HB information. None of the individual tests show significant results for *CYP4F3* in any population. *CYP4F12* shows iHS values slightly above 2 sd of the genome-wide mean in most African populations (Additional file 1: Figure S9a), and the F_ST_ comparison between CEU and CHB populations also has a high value (above 2 sd) (Additional file 1: Figure S9b). However, we cannot conclude that *CYP4F12* has been under positive selection due to the lack of information in HB.

Thus, even if using single neutrality tests some genic regions may show extreme values, the use of a composite method does not give a statistically significant result in any of them. Therefore, our results suggest that positive selection has not acted on *CYP1*, *CYP2* and *CYP4* genes in any of the studied populations.

#### Positive selection has acted on CYP3 locus

**Fig. 1:**
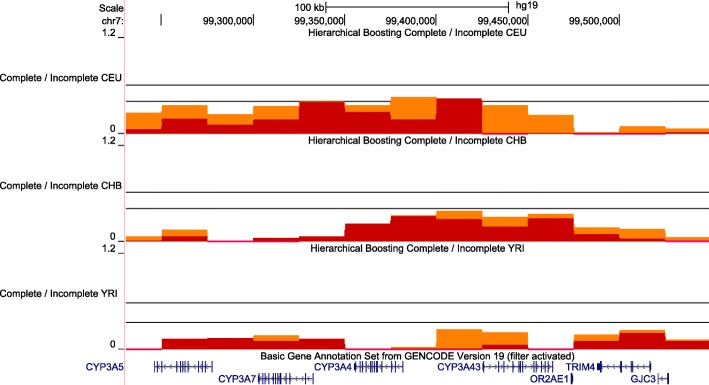
Hierarchical boosting scores for the *CYP3A* locus: *CYP3A4*, *CYP3A5*, *CYP3A7* and *CYP3A43*. Results for CEU, CHB and YRI populations are shown for both Complete (in red) and Incomplete boosting (in orange). There is a complete selective sweep in the promoter area shared by *CYP3A4* and *CYP3A43* in CEU. Black lines indicate genome-wide significance thresholds (False Discovery Rate, FDR 1%). For the Complete/Incomplete tracks the higher threshold corresponds to Incomplete scores and the lower to Complete

In contrast, HB revealed significant signatures of positive selection in the *CYP3* loci in CEU. Specifically, HB shows statistically significant scores for a complete selective sweep in *CYP3A4* and *CYP3A43* promoter regions and an almost significant signal of a complete selective sweep in *CYP3A7* (Fig. 1).

In addition to a significant HB score at the *CYP3A4* promoter region, the coding region showed significant deltaDAF scores in CEU when compared with YRI (Additional file 1: Figure S10a). The PopHuman Phase 3 data also displayed significant results in the *CYP3A4* coding region for XP-EHH comparison of CEU and CHB with YRI as well as F_ST_ for CEU comparison with YRI (Additional file 1: Figure S10b-c).

The other gene of the family with a clear complete selective sweep in CEU, *CYP3A43*, also shows in the coding region high F_ST_ values when comparing CEU and CHB with YRI in the PopHuman Phase 3 data (above 2 sd) (Additional file 1: Figure S10c).

When looking at signals of selection in *CYP3A7*, the HB score is close to reaching statistical significance in CEU (Fig. 1). PopHuman Browser Phase 3 displays a strong signal from XP-EHH when comparing CEU and CHB with YRI (higher than 2 sd from the mean) (Additional file 1: Figure S10b). In addition, we also find significant iHS scores in some European and in all Asian populations (Additional file 1: Figure S10d).

As for *CYP3A5*, we did not find evidence of positive selection in the HB. But Phase 1 data XP-CLR (Additional file 1: Figure S10a) and Phase 3 data XP-EHH revealed significant results when comparing CEU with YRI (Additional file 1: Figure S10b). These last results using only individual selection tests from Phase 1 and 3 data explain why Li et al. [44] claimed that selection acted in the *CYP3A5* locus, contrarily to our results.

Hence, there is strong evidence of positive selection in *CYP3A4* and *CYP3A43* in CEU, while further evidence would be needed to confirm that natural selection acted on the *CYP3A7* locus.

#### CYP27A1 as a new gene candidate under positive selection in Asia

Finally, we analyzed whether *CYP* genes from other families that might play minor roles in biotransformation, have also been under positive selection (Table 1).

**Fig. 2:**
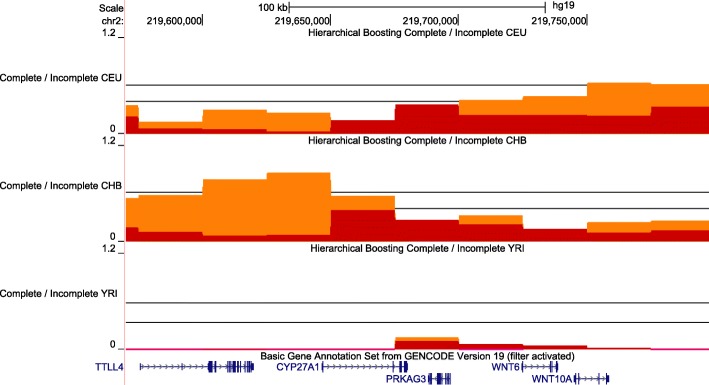
Hierarchical boosting scores for the *CYP27A1* gene. Results for CEU, CHB and YRI populations are shown for both Complete (in red) and Incomplete boosting (in orange). There is an incomplete selective sweep in CHB at the beginning of *CYP27A1*. Black lines indicate genome-wide significance thresholds (FDR 1%). For the Complete/Incomplete tracks the higher threshold corresponds to Incomplete scores and the lower to Complete

Only *CYP27A1*, a gene that transforms cholesterol into bile acids and participates in the synthesis of vitamin D3, showed signals of an incomplete selective sweep in CHB (Fig. 2). In PopHuman Phase 3, *CYP27A1* has no Tajima’s D information. Haplotype-based tests (XP-EHH) are significant when comparing CEU and CHB with YRI (Additional file 1: Figure S11a). F_ST_ comparison is the main driver of the signal, with both comparisons of CHB with CEU and YRI having values above 2 sd of the genome-wide mean (Additional file 1: Figure S11b).

## Discussion

The analysis conducted in this study showed that none of the known taste receptor genes have been under recent positive selection in any of the populations considered. It is important to note that the individual tests used in previous studies that detected positive selection in some of these genes are less powerful and precise [55–57]. One of the arguments to differentiate selective sweeps from the effect of demographic forces is that positive selection is a local event, whereas demographic events have a global effect on the genome. However, these local signatures can be amplified by the action of demographic events, thus generating a signal compatible with a selection event [58, 67]. To reduce false positives, we relied on a genome-wide scan of positive selection by a composite method (HB) that integrates several selection and neutrality tests and takes population demography into account [54].

The results of HB contradict previous studies that suggested that the whole *TAS1R* gene family has been under positive selection [10, 11].

For the *TAS2R* gene family, our results support a previous finding: bitter taste receptors overall are not under positive selection in humans [22]. In some cases, such as *TAS2R16*, our findings agree with previous studies that hypothesized that taste receptor genes evolved at a very early stage of human evolution and had not been positively selected after the Out of Africa expansion [20]. Similarly, we did not observe signs of positive or balancing selection in *TAS2R38*, in agreement with a previous study that suggested that variation in this gene was due to genetic drift [16, 22].

In addition, salty and sour taste receptors did not show signatures of positive selection (except *SCNN1D* that has inconclusive results).

Overall, our results suggest that positive selection has not been acting on taste receptor genes during recent human evolution. These genes are involved in other processes beyond taste and might be more functionally restrained. Thus, differences among human populations in the physiology of taste are caused by drift, as no new adaptations have emerged in the process of diversification of human populations.

Regarding the *CYP450* system, genes in *CYP1* and *CYP2* families that were previously reported to be under positive selection [43–46] did not show any signatures of selection in our results. Therefore, no specific adaptations have emerged related to *CYP1* and *CYP2* over the recent history of modern humans.

We do find signatures of selective sweeps in two genes belonging to the *CYP3* family: *CYP3A4* and *CYP3A43* promoter regions are under positive selection in CEU. This result is partially in agreement with a previous study where signals of selection were found in all populations [51]. Nevertheless, that study did not correct their results for the confounding effects of demography and hence they might have a higher rate of false positives. With the HB information, it was possible to further classify this selection as complete, meaning that the substitution has reached fixation. These results point towards an important regulatory adaptation in CEU in *CYP3A4.* Given the central role of *CYP3A4* in drug metabolism, these findings could help develop a better understanding of the interpopulation differences in xenobiotic clearance. However, most *CYP* enzymes have more than one function. Even if the main function of *CYP3A4* and *CYP3A43* is biotransformation, their other physiological functions (such as the synthesis of steroid hormones) could also be potentially important in increasing survival fitness [68].

Moreover, we identify a previously unreported *CYP* gene under positive selection. HB revealed a strong selective sweep in *CYP27A1* in CHB. *CYP27A1* is mainly expressed in liver and intestine, and participates in bile acid biosynthesis and vitamin D_3_ metabolism [69], not in biotransformation [38, 52]. Arciero et al. found signals of positive selection in East Asians populations in genes associated with vitamin D action in bones, but not in vitamin D synthesis pathway, where CYP27A1 participates [70]. It is very likely that the phenotype under selection associated with CYP27A1 is not related to the transformation of xenobiotics but to vitamin D metabolism.

## Conclusions

We used a robust and uniform method to detect selective sweeps in all known genes related to taste perception and phase I biotransformation (CYP450 system). This study supports the hypothesis that genes involved in taste perception have not been positively selected in recent human evolution. Thus, different allele frequencies between human populations in genes such as *TAS2R38* and *TAS2R16* are not due to positive selection but to genetic drift. Moreover, we detected in CEU signatures of positive selection in the promoter region of *CYP3A4,* which plays a key role in drug metabolism. This could indicate a regulatory adaptation in the expression of *CYP3A4*. Finally, another *CYP* gene, *CYP27A1*, which participates in the metabolism of cholesterol and vitamin D3, showed signatures of positive selection in CHB.

## Methods

A total of 91 genes related to taste perception and phase I biotransformation were selected to study signals of positive selection (Additional file 2: Table S1): 34 taste receptor genes, and 57 genes encoding for cytochrome enzymes.

To identify whether positive selection has acted on them, each gene region was investigated in a database of hard selective sweeps in human populations [54]. This dataset was generated by applying a hierarchical classification framework based on a boosting algorithm (Hierarchical Boosting or HB) to three continental populations from the 1000 Genomes Project Phase 1: Yoruba from Ibadan, Nigeria (YRI); Han Chinese from Beijing, China (CHB); and Utah residents with Northern and Western European Ancestry (CEU). It was selected as it is calculated through the combination of several tests of positive selection [65], thus providing information about which signature of positive selection underlies the signal observed. In addition, it classifies events of positive selection as Complete or Incomplete depending on whether the selected variant is fixed or not.

In order to get a more detailed view, specific neutrality tests and selection statistics have been used (Additional file 2: Table S2; Additional file 1: Supplementary Note), either from the 1000 Genomes Selection Browser 1.0 for Phase 1 [65], or the PopHuman Browser for Phase 3 [66]. We selected statistics based on population differentiation (deltaDAF, F_ST_ and XP-CLR) and linkage disequilibrium structure (iHS and XP-EHH) to identify genes under positive selection, and a site frequency spectrum-based test (Tajima’s D), to identify genes under positive or balancing selection. Statistics from the 1000 Genomes Selection Browser 1.0 for Phase 1 were considered statistically significant if the rank score (genome-wide based ranked *p*-values) was higher than 2 (False Discovery Rate, FDR 1%). In the case of the PopHuman Browser, as it is not possible to obtain the statistical significance of the signals, values where compared to the genome-wide mean ± 2 standard deviations (sd).

## Additional file

Additional file 1: Supplementary Note. Brief description of the three browsers used. **Figure S1.** Hierarchical boosting scores for well-known cases of genes under positive selection. **Figure S2.** Selection tests from the 1000 Genomes Selection Browser 1.0 in the *TAS1R3* gene. **Figuye S3.** Selection tests in the *TAS2R16* and *TAS2R38* genes. **Figure S4.** Hierarchical boosting scores for sour tasting related genes. **Figure S5.** Hierarchical boosting scores for salty tasting related genes. **Figure S6.** Hierarchical boosting scores for *CYP1A2* gene. **Figure S7.** Hierarchical boosting scores and selection tests from PopHuman Browser Phase 3 for *CYP2E1* gene. **Figure S8.** Hierarchical boosting scores and selection tests from PopHuman Browser Phase 3 for *CYP4B1* and *CYP4Z1*. **Figure S9.** Selection tests from PopHuman Browser Phase 3 for *CYP4F12*. **Figure S10.** Selection tests from the 1000 Genomes Selection Browser 1.0 and PopHuman Browser Phase 3 in the CYP3 locus. **Figure S11.** Selection tests from PopHuman Browser Phase 3 in the *CYP27A1* gene. (PDF 2090 kb)

Additional file 2: **Table S1.** Genes related to taste perception and biotransformation phase I analyzed in this study. **Table S2.** Selection tests from the 1000 Genomes Selection Browser 1.0 [54] and PopHuman [66] inspected in this study (XLSX 25 kb)

## Abbreviations

CEU: Utah residents with Northern and Western European Ancestry
CHB: Han Chinese from Beijing, China
FDR: False Discovery Rate
HB: Hierarchical Boosting
PTC: Phenylthiocarbamide
sd: standard deviation
YRI: Yoruba from Ibadan, Nigeria
