# Supplementary material for: Is there adaptation in the human genome for taste perception and phase I biotransformation?
Source: BMC Evol Biol. 2019 Jan 31;19:39. doi: 10.1186/s12862-019-1366-7 (PMC6357387; doi:10.1186/s12862-019-1366-7)
Supplement: Supplementary file 1 — Supplementary Note. Brief description of the three browsers used. Figure S1. Hierarchical boosting scores for well-known cases of genes under positive selection. Figure S2. Selection tests from the 1000 Genomes Selection Browser 1.0 in the TAS1R3 gene. Figuye S3. Selection tests in the TAS2R16 and TAS2R38 genes. Figure S4. Hierarchical boosting scores for sour tasting related genes. Figure S5. Hierarchical boosting scores for salty tasting related genes. Figure S6. Hierarchical boosting scores for CYP1A2 gene. Figure S7. Hierarchical boosting scores and selection tests from PopHuman Browser Phase 3 for CYP2E1 gene. Figure S8. Hierarchical boosting scores and selection tests from PopHuman Browser Phase 3 for CYP4B1 and CYP4Z1. Figure S9. Selection tests from PopHuman Browser Phase 3 for CYP4F12. Figure S10. Selection tests from the 1000 Genomes Selection Browser 1.0 and PopHuman Browser Phase 3 in the CYP3 locus. Figure S11. Selection tests from PopHuman Browser Phase 3 in the CYP27A1 gene. (PDF 2090 kb) [file 12862_2019_1366_MOESM1_ESM.pdf]

SUPPLEMENTARY NOTE

Brief description of the UCSC tracks from the three browsers used:

1000 Genomes Selection Browser 1.0

The 1000 Genomes Selection Browser 1.0 [1] shows a UCSC track for each neutrality and selection test calculated for each population. The tracks show “rank scores”, which are genome-wide based ranked “p-values”. For each test in each population, Pybus et al. (2015) sorted the scores from highest to lowest and calculated the rank score as the fraction of values that are higher than a given score in the genome-wide distribution. Then, they converted the rank score to -log10(rank score), meaning that a region with a p-value higher than 2 has a rank score of 0.01 and is significant at a 1% False Discovery Rate (FDR 1%). As there are several neutrality and selection tests calculated in the Selection Browser 1.0, we focused on 6 tests for comparison with previous studies that analyzed positive or balancing selection on taste receptor genes or cytochrome P450 genes: F_{ST} [2], deltaDAF (difference of Derived Allele Frequencies between two populations), iHS [3], Tajima’s D [4], XP-EHH [5], and XP-CLR [6]. All tests were applied to the three continental populations (CEU, CBH and YRI) from 1000 Genomes Project Phase 1.

Hierarchical Boosting

The Hierarchical Boosting browser [7] shows a UCSC track for each population combining the results of the Complete boosting (in red) and the Incomplete boosting (in orange). Each test has a genome-wide significance threshold indicated at FDR 1% by a black line. The higher threshold belongs to the Incomplete boosting score (in orange) and the lower threshold to the Complete boosting score (in red). The Hierarchical Boosting (HB) was applied to the three continental populations (CEU, CHB and YRI) from 1000 Genomes Project Phase 1.

PopHuman Browser Phase 3

The PopHuman Browser Phase 3 [8] shows a UCSC track for each neutrality and selection test calculated for each population. The tracks show the raw scores. For each test in each population, Casillas et al. (2018) calculated the genome-wide mean of the test (yellow line) and the standard deviation (sd). The dark grey lines indicated ± one sd from the genome-wide mean, while the light grey lines indicate ± 2 sd from the genome-wide mean. In this case, the authors did not calculate p-values, and we used as a threshold of significance the score indicating ± 2 sd from the genome-wide mean. As with the Selection Browser 1.0, we focused on 6 tests for comparison with previous studies: F_{ST} [9], deltaDAF, iHS, Tajima’s D, and XP-EHH. For Tajima’s D, positive scores (balancing selection) are indicated in green, whereas negative scores (positive selection) are indicated in red. Pairwise comparisons (F_{ST} and XP-EHH) were only computed using the three continental populations: CEU, CHB and YRI. The other tests were applied to the 26 populations of 1000 Genomes Project Phase 3, but we removed from our analysis admixed American populations (MXL, PUR, CLM, PEL) and African-American (ASW), as their complex ancestry can affect the results of the selection tests.

SUPPLEMENTARY FIGURES

Figure S1. Hierarchical boosting scores for well-known cases of genes under positive selection. a) Complete selective sweep in the SLC24A5 loci in CEU. b) Complete selective sweep in the LCT loci in CEU. c) Incomplete selective sweep in EDAR in CHB. d) Complete selective sweep in DARC in YRI. Results for CEU, CHB and YRI are shown for both Complete boosting (red) or Incomplete (orange). Black lines indicate genome-wide significance thresholds (False Discovery Rate, FDR 1%). For the Complete/Incomplete tracks the higher threshold corresponds to Incomplete scores and the lower to Complete. Significant scores are highlighted by a red square, gene region is delimitated by a black square.

a)

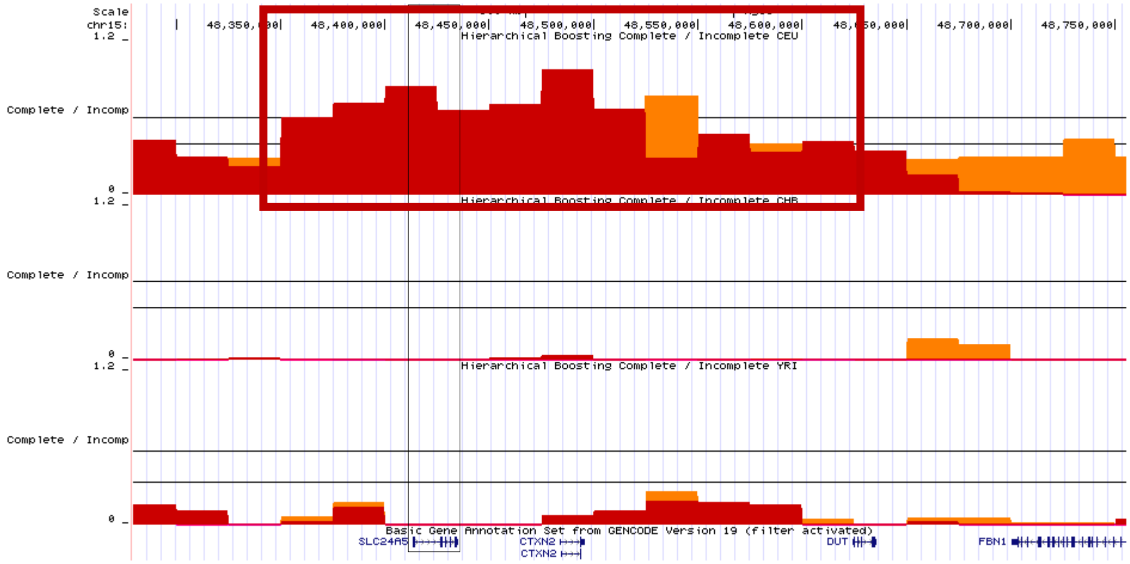

b)

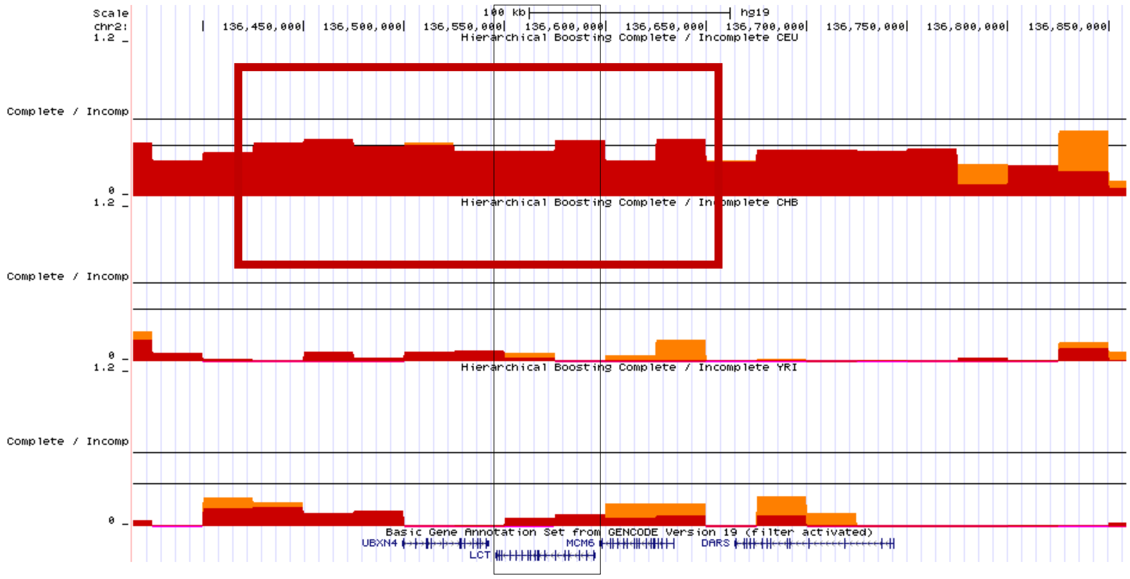

c)

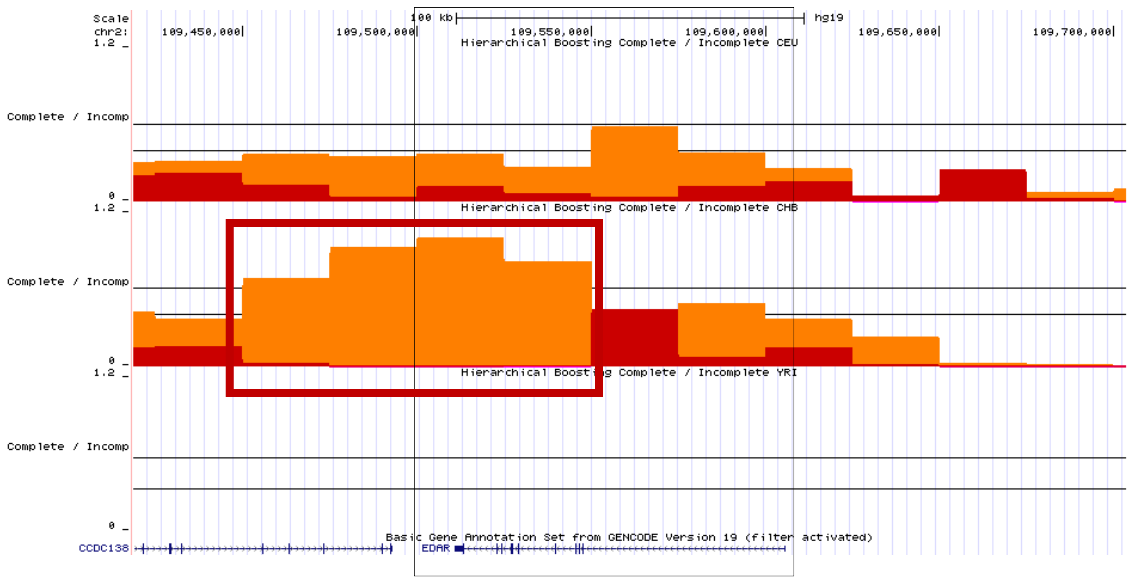

Scale
chr2:
100 kb
hg19
109,450,000|
109,500,000|
109,550,000|
109,600,000|
109,650,000|
109,700,000|
Hierarchical Boosting Complete / Incomplete CEU
Complete / Incomp
Hierarchical Boosting Complete / Incomplete CHB
Hierarchical Boosting Complete / Incomplete YRI
Basic Gene Annotation Set from GENCODE Version 19 (filter activated)
CCDC138
EDAR

d)

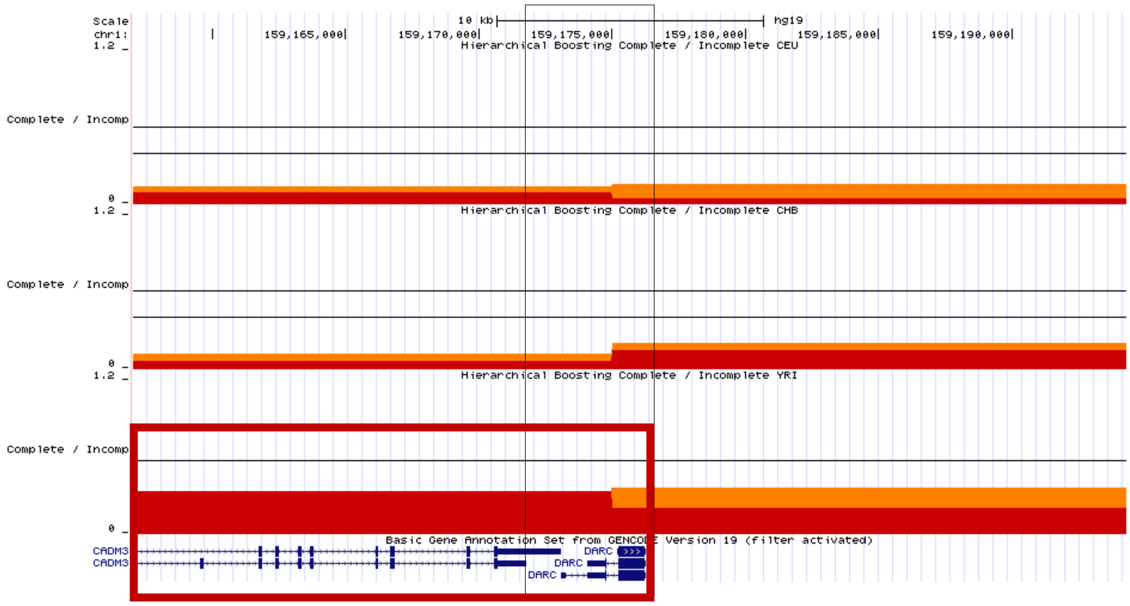

Scale
chr1:
10 kb
hg19
159,165,000|
159,170,000|
159,175,000|
159,180,000|
159,185,000|
159,190,000|
Hierarchical Boosting Complete / Incomplete CEU
Complete / Incomp
Hierarchical Boosting Complete / Incomplete CHB
Hierarchical Boosting Complete / Incomplete YRI
Basic Gene Annotation Set from GENCODE Version 19 (filter activated)
CADM3
DARC

Figure S2. Selection tests from the 1000 Genomes Selection Browser 1.0 in the *TAS1R3* gene. DeltaDAF tracks comparing the three continental populations show high population differentiation in CEU in the promoter area of *TAS1R3*. When comparing CEU with CHB, only one SNP reaches the threshold of significance at FDR 1%, whereas two SNPs are significant when comparing CEU with YRI. Significant scores are highlighted by a red square, gene region is delimitated by a black square.

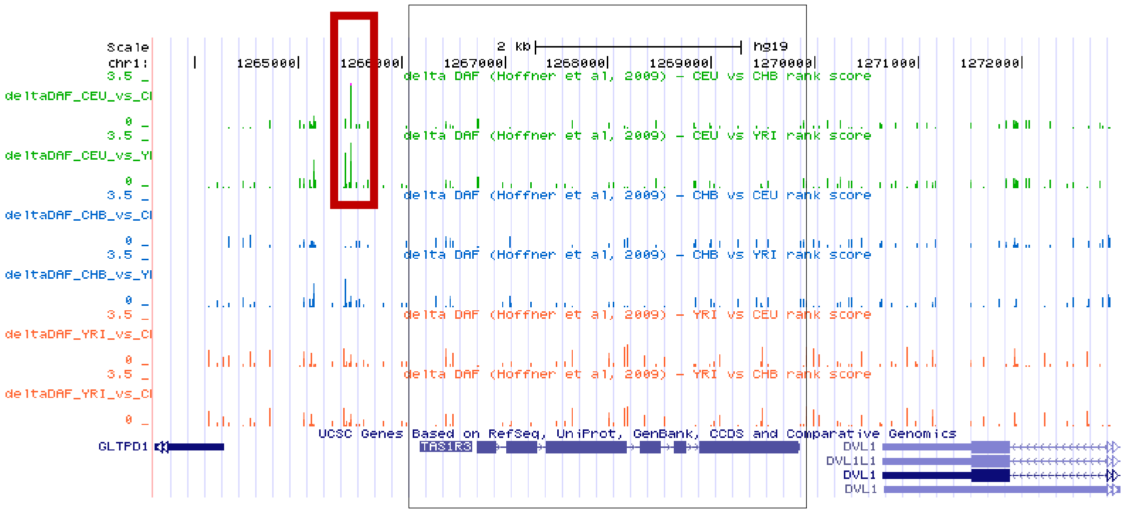

Figure S3. Selection tests in the *TAS2R16* and *TAS2R38* genes. a) DeltaDAF tracks when comparing YRI with CEU and CHB show one statistically significant SNP in *TAS2R16* (there is no information for Phase 3 populations in PopHuman). b) 1000 Genomes Selection Browser 1.0 shows no signatures of balancing or positive selection in *TAS2R38* in any of the three continental populations (CEU, CHB and YRI). c) PopHuman Browser Phase 3 shows no evidence of balancing or positive selection in *TAS2R38* in none of the populations. Here we show the results for the three continental populations (CEU, CHB and YRI), but we have added a link to the information of the other 21 populations. Positive Tajima's D scores (green) indicate balancing selection, whereas negative scores (red) indicate positive selection. Significant scores are highlighted by a red square, gene region is delimitated by a black square.

a)

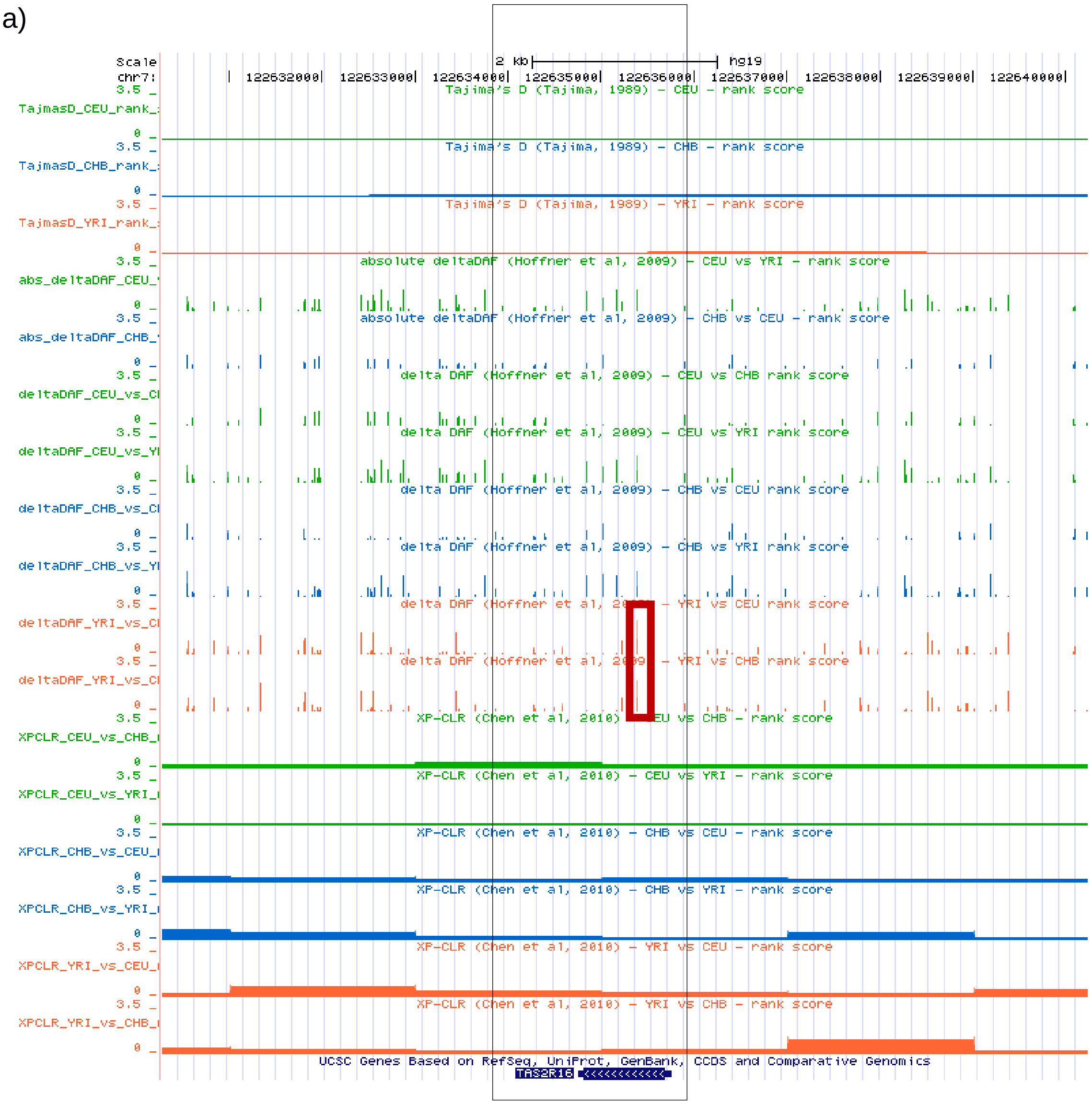

b)

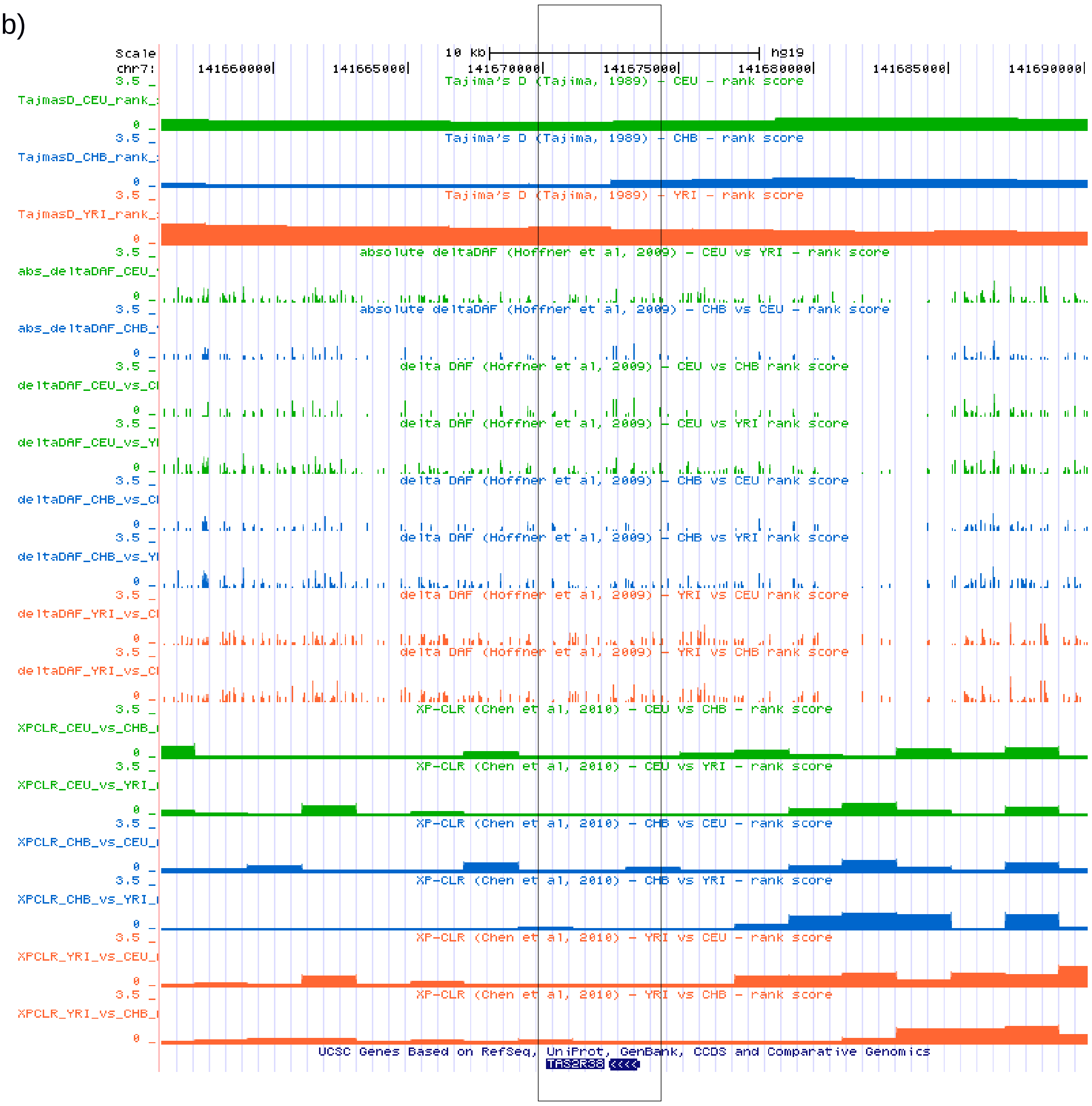

c)

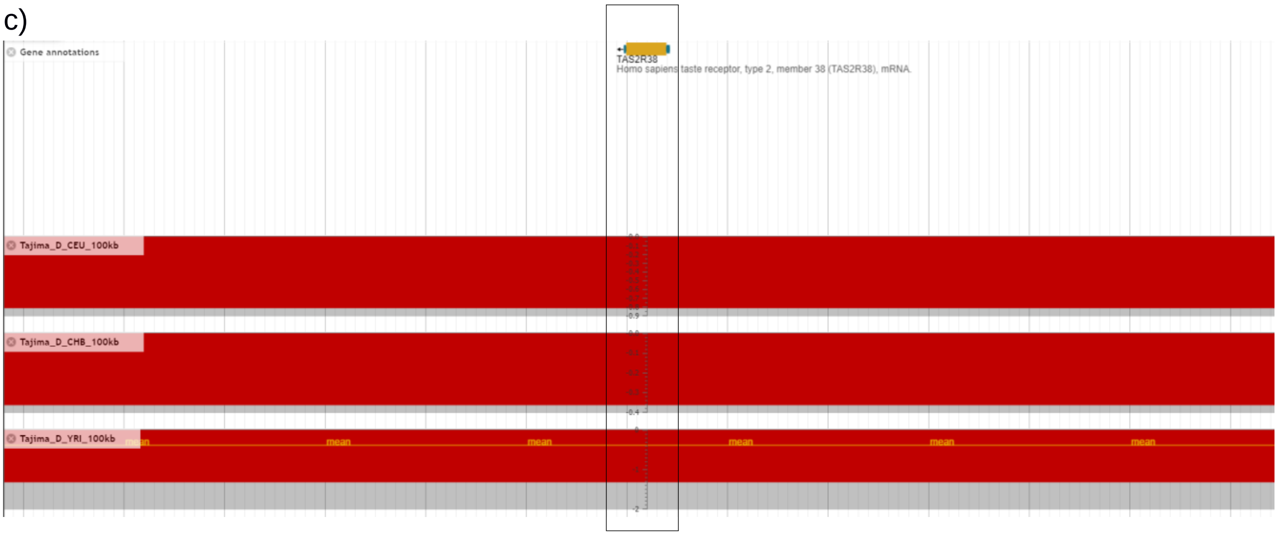

[Tajima'D scores in TAS2R38 in the rest of populations from 1000 Genomes Project Phase 3]

Figure S4. Hierarchical boosting scores for sour tasting related genes. a) No evidence of positive selection in *PKD2L1* gene in any of the three continental populations. b) No evidence of positive selection in *PKD1L3* gene in any of the three continental populations. c) No evidence of positive selection in *OTOP1* gene in any of the three continental populations. Results for CEU, CHB and YRI are shown for both Complete boosting (in red) and Incomplete (in orange). Black lines indicate genome-wide significance thresholds (FDR 1%). The higher threshold corresponds to Incomplete scores and the lower to Complete HB. Gene region is delimitated by a black square.

a)

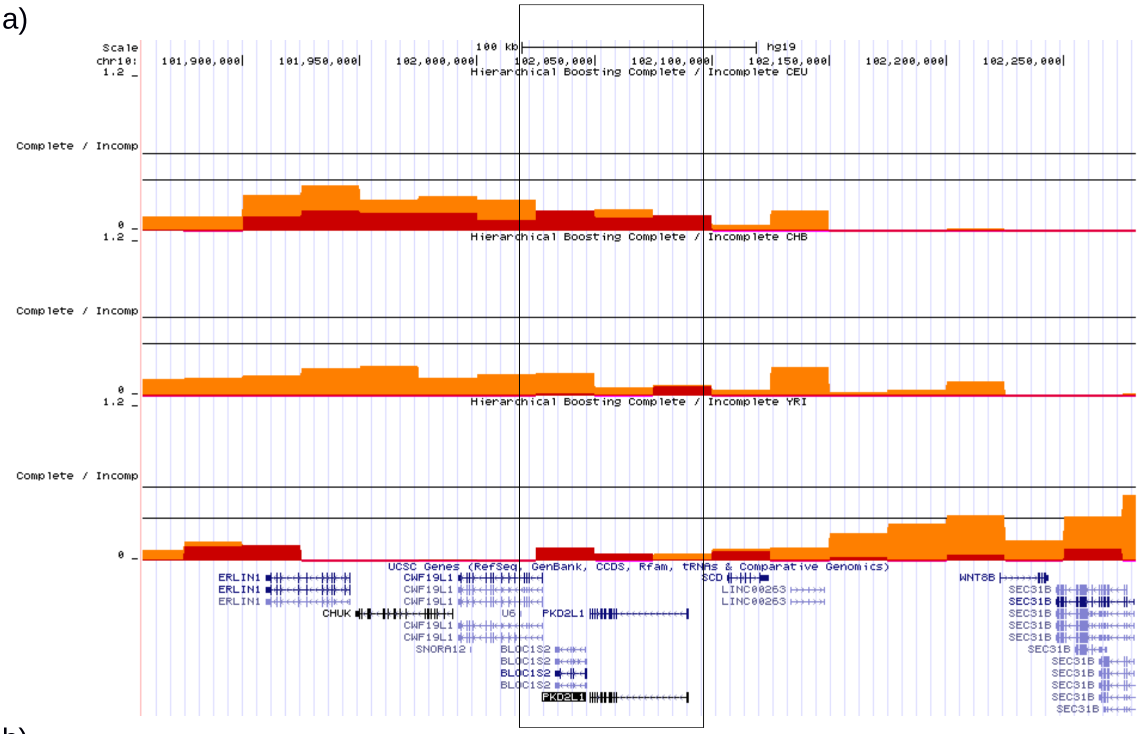

b)

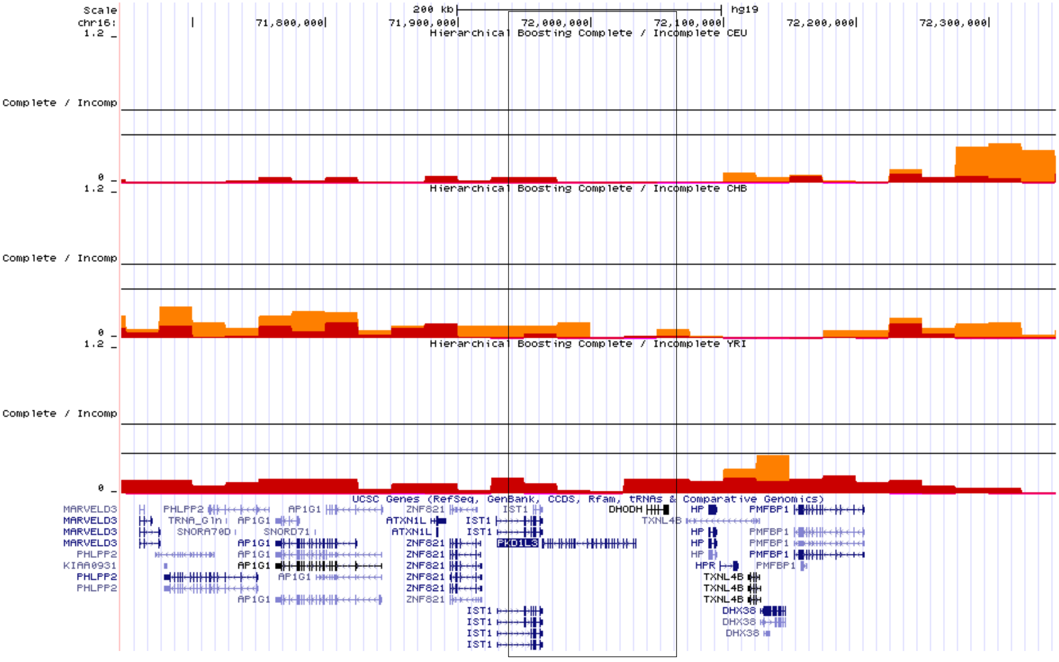

c)

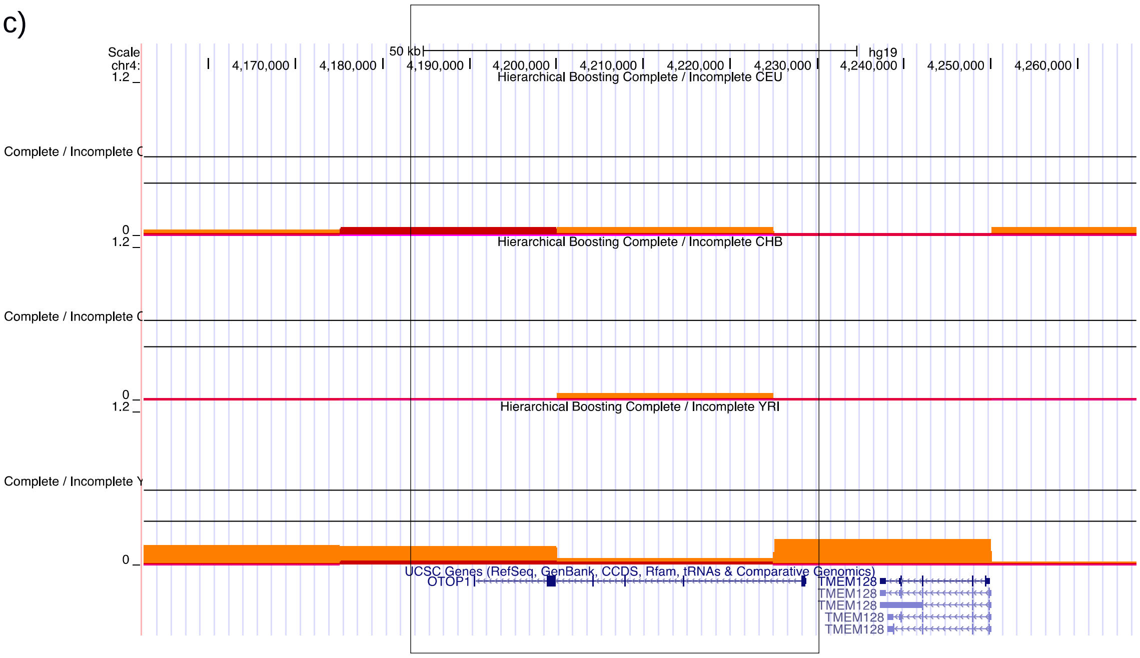

Scale
50 kb
hg19
chr4:
4,170,000
4,180,000
4,190,000
4,200,000
4,210,000
4,220,000
4,230,000
4,240,000
4,250,000
4,260,000
1.2
Hierarchical Boosting Complete / Incomplete CEU
Complete / Incomplete C
0
1.2
Hierarchical Boosting Complete / Incomplete CHB
Complete / Incomplete C
0
1.2
Hierarchical Boosting Complete / Incomplete YRI
Complete / Incomplete Y
0
UCSC Genes (RefSeq, GenBank, CCDS, Rfam, tRNAs & Comparative Genomics)
OTOP1
TMEM128
TMEM128
TMEM128
TMEM128
TMEM128

Figure S5. Hierarchical boosting scores for salty tasting related genes. a) No evidence of positive selection in *SCNN1A* gene in any of the three continental populations. b) No evidence of positive selection in *SCNN1B* and *SCNN1G* genes in any of the three continental populations. Results for CEU, CHB and YRI are shown for both Complete boosting (in red) and Incomplete (in orange). Black lines indicate genome-wide significance thresholds (FDR 1%). The higher threshold corresponds to Incomplete scores and the lower to Complete HB. c) Selection tests from 1000 Genomes Selection Browser 1.0 for *SCNN1D*. Few SNPs show significant scores when comparing CEU with CHB or YRI in deltaDAF and in XP-CLR when comparing CEU with CHB or YRI. Significant scores are highlighted by a red square, gene region is delimitated by a black square.

a)

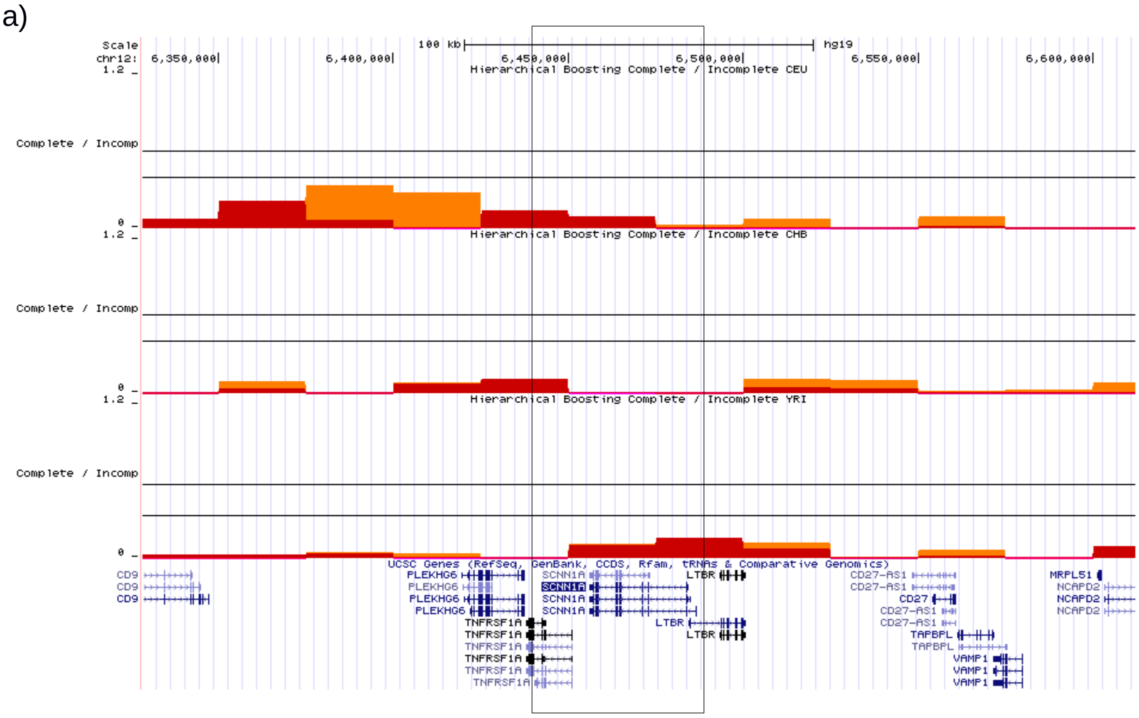

b)

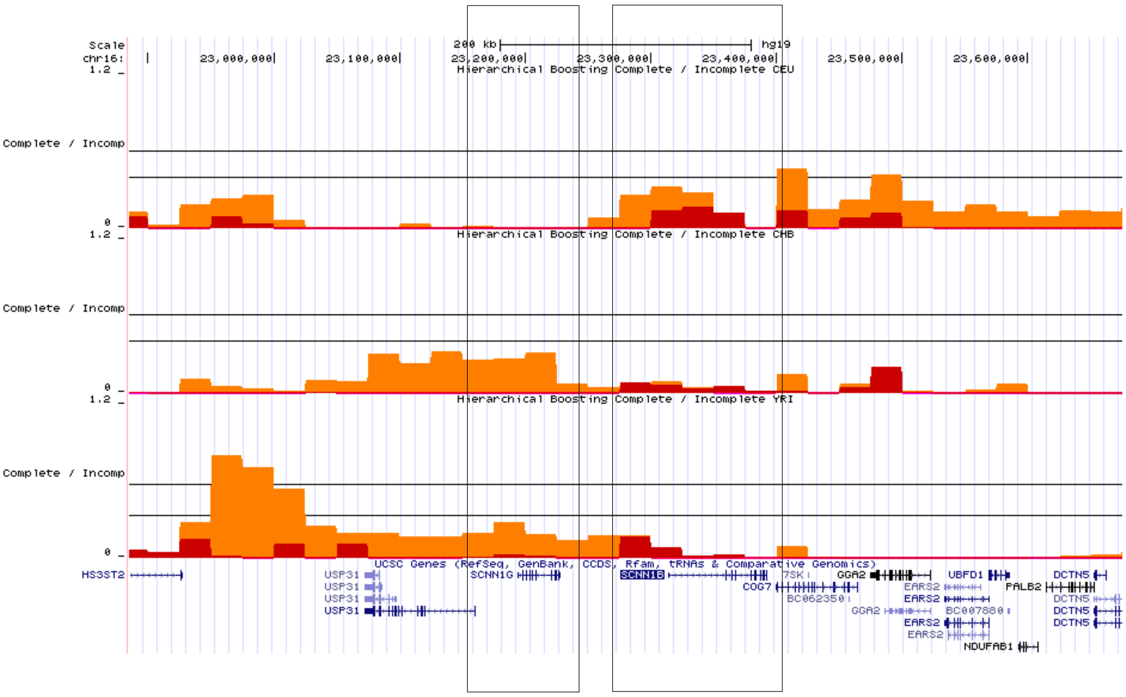

c)

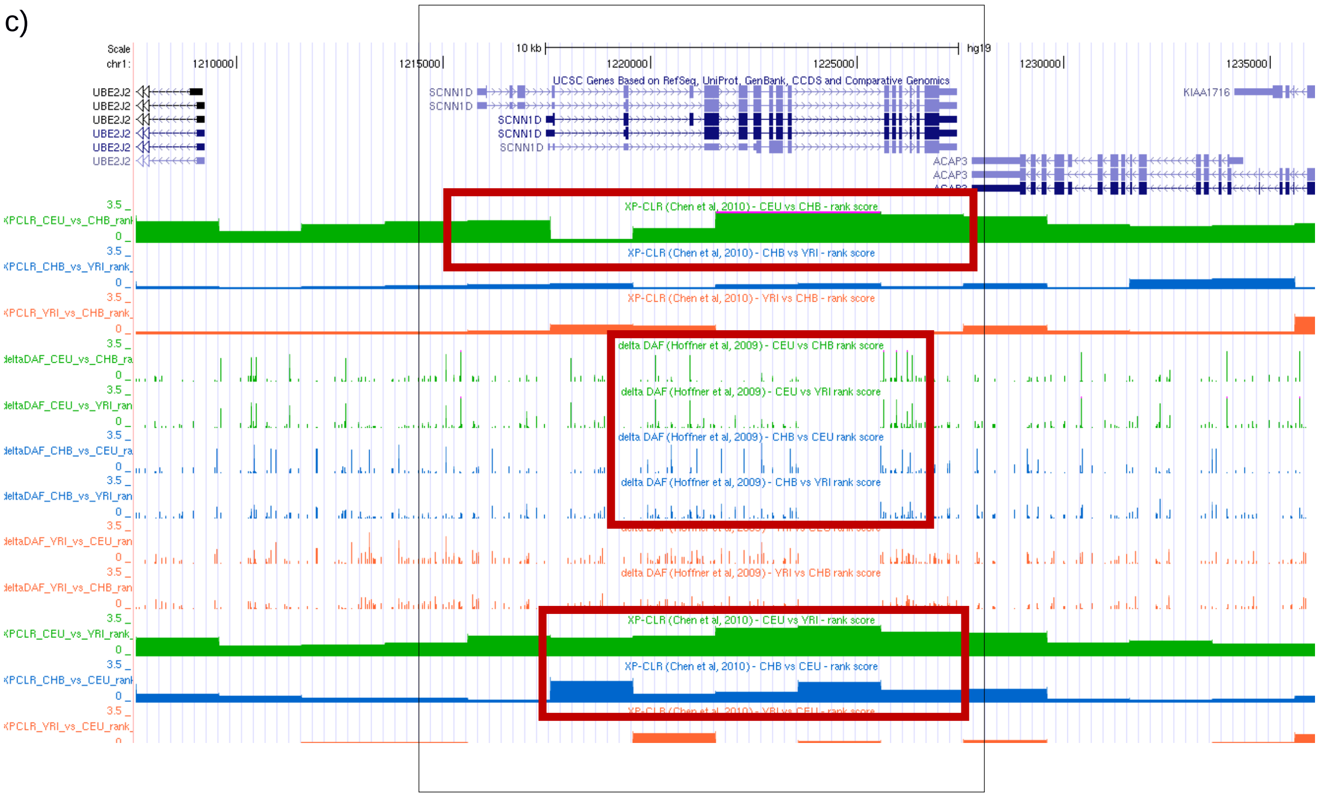

Scale
chr1:
10 kb
hg19
1210000
1215000
1220000
1225000
1230000
1235000
UCSC Genes Based on RefSeq, UniProt, GenBank, CCDS and Comparative Genomics
UBE2J2
SCNN1D
KIAA1716
ACAP3
XP-CLR (Chen et al, 2010) - CEU vs CHB - rank score
XP-CLR (Chen et al, 2010) - CHB vs YRI - rank score
XP-CLR (Chen et al, 2010) - YRI vs CHB - rank score
delta DAF (Hoffner et al, 2009) - CEU vs CHB rank score
delta DAF (Hoffner et al, 2009) - CEU vs YRI rank score
delta DAF (Hoffner et al, 2009) - CHB vs CEU rank score
delta DAF (Hoffner et al, 2009) - CHB vs YRI rank score
delta DAF (Hoffner et al, 2009) - YRI vs CHB rank score
XP-CLR (Chen et al, 2010) - CEU vs YRI - rank score
XP-CLR (Chen et al, 2010) - CHB vs CEU - rank score
3.5
0

Figure S6. Hierarchical boosting scores for *CYP1A2* gene. No evidence of positive selection is found in any of the three continental populations. Results for CEU, CHB and YRI are shown for both Complete boosting (red) or Incomplete (orange). Black lines indicate genome-wide significance thresholds (FDR 1%). For the Complete/Incomplete tracks the higher threshold corresponds to Incomplete scores and the lower to Complete. Gene region is delimitated by a black square.

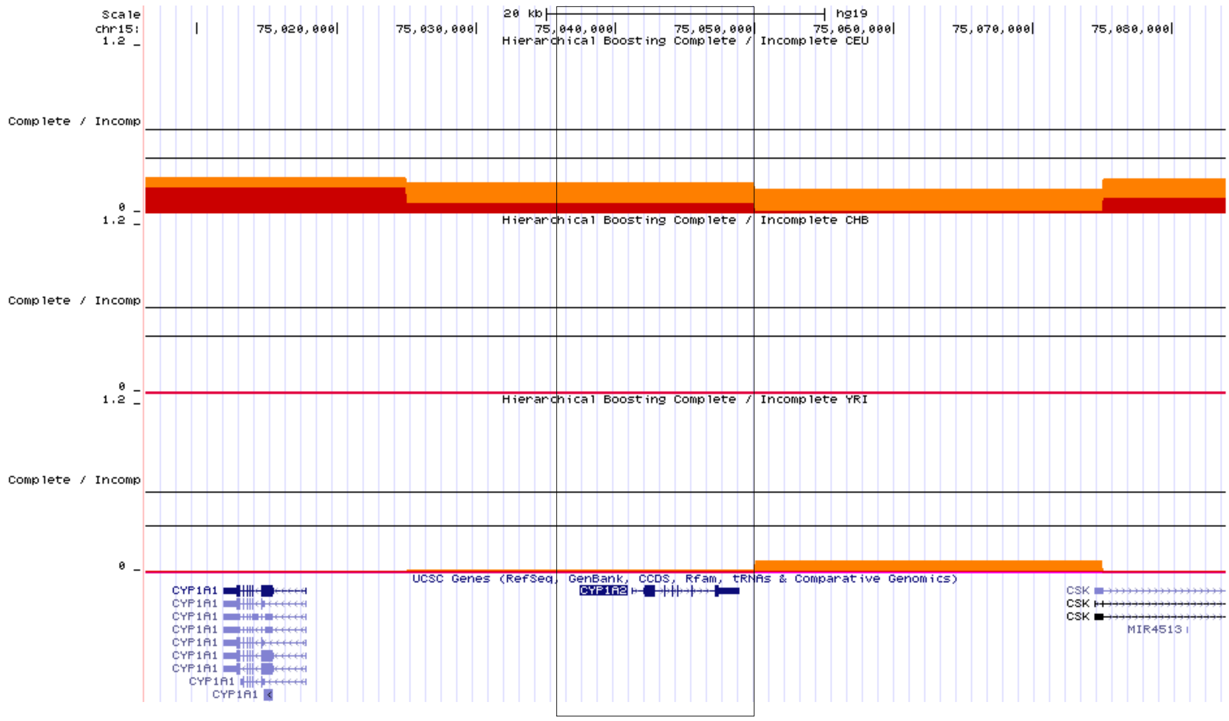

Figure S7. Hierarchical boosting scores and selection tests from PopHuman Browser Phase 3 for *CYP2E1* gene. a) F_{ST} and XP-EHH tracks show scores above 2 standard deviations (sd) from the genome-wide mean when comparing CEU with YRI, as well as the iHS values of CEU and YRI. b) However, HB scores do not show evidence of positive selection in any of the three continental populations. Results for CEU, CHB and YRI are shown for both Complete boosting (red) or Incomplete (orange). Black lines indicate genome-wide significance thresholds (FDR 1%). For the Complete/Incomplete tracks the higher threshold corresponds to Incomplete scores and the lower to Complete. Significant scores are highlighted by a red square, gene region is delimitated by a black square.

a)

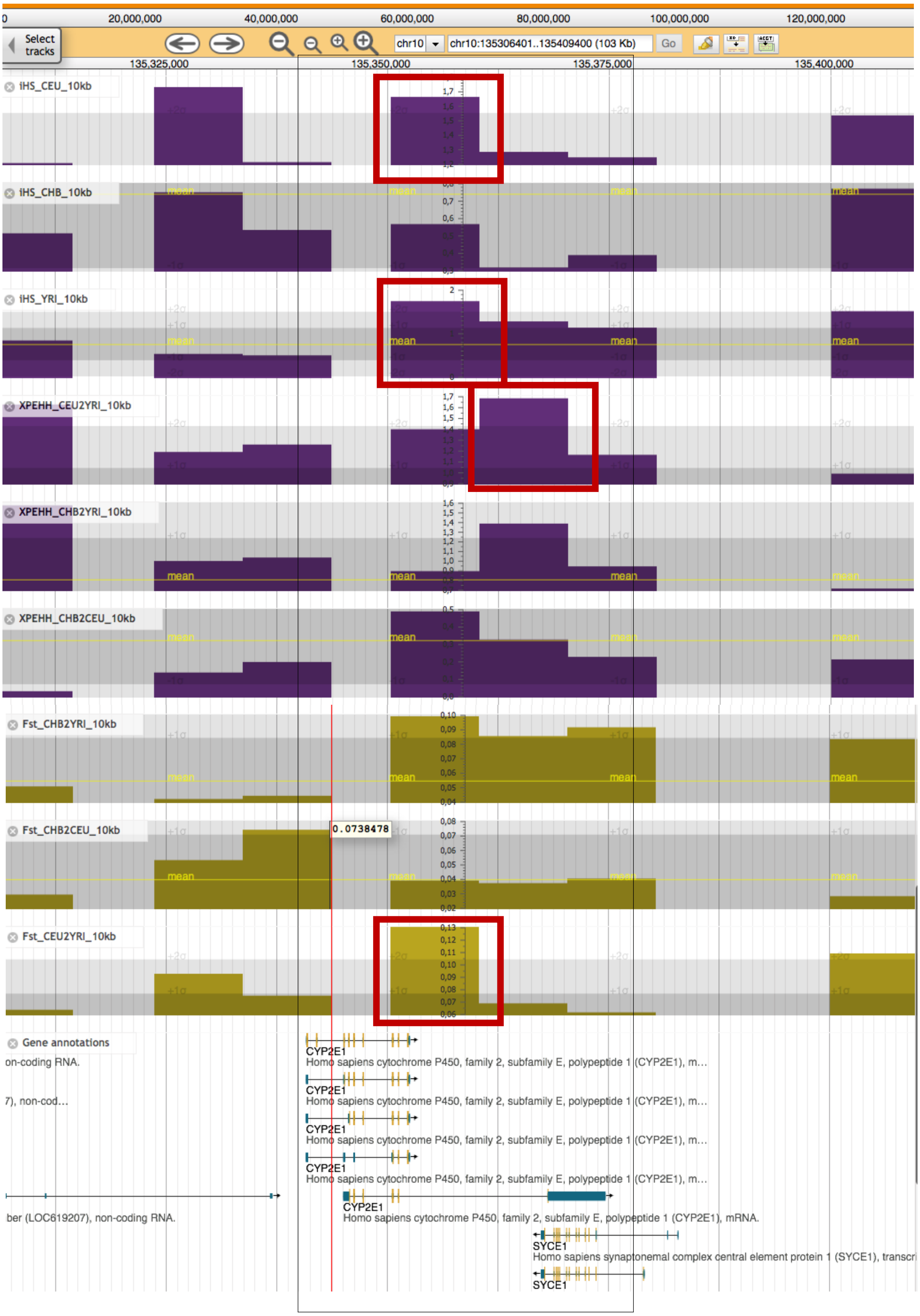

b)

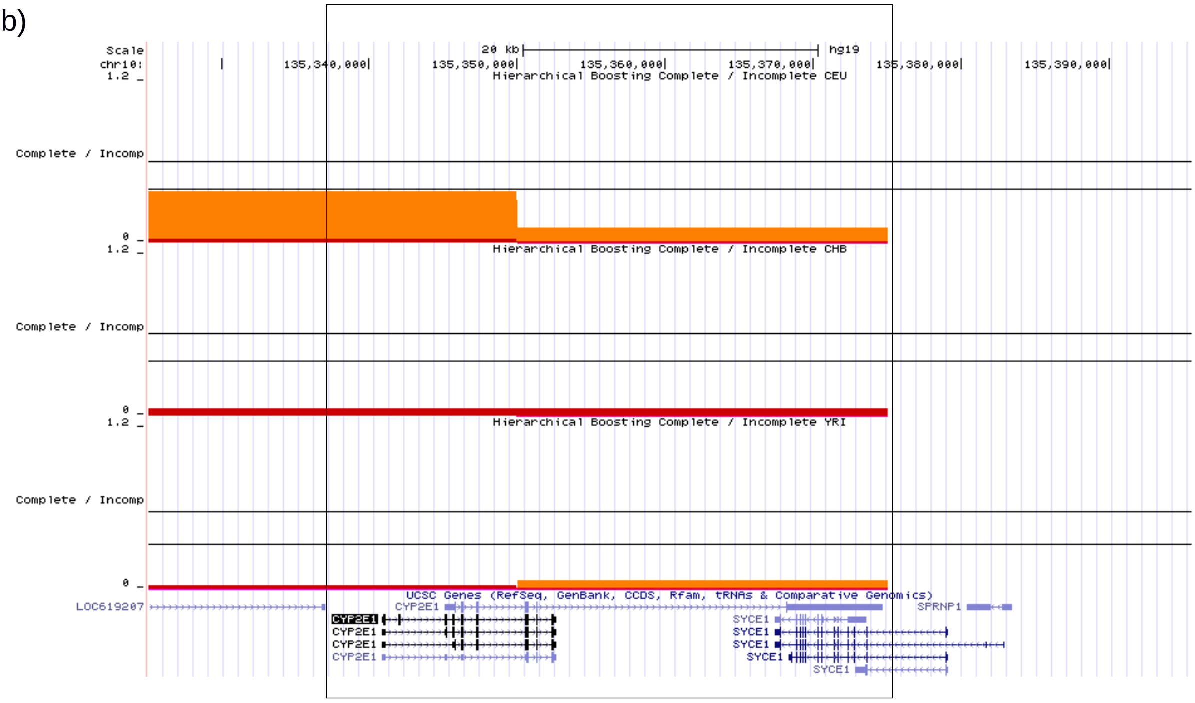
Scale
chr10:
20 kb
hg19
135,340,000
135,350,000
135,360,000
135,370,000
135,380,000
135,390,000
Hierarchical Boosting Complete / Incomplete CEU
1.2 _
Complete / Incomp
0 _
Hierarchical Boosting Complete / Incomplete CHB
1.2 _
Complete / Incomp
0 _
Hierarchical Boosting Complete / Incomplete YRI
1.2 _
Complete / Incomp
0 _
UCSC Genes (RefSeq, GenBank, CCDS, Rfam, tRNAs & Comparative Genomics)
LOC619207
CYP2E1
CYP2E1
CYP2E1
CYP2E1
CYP2E1
SYCE1
SYCE1
SYCE1
SYCE1
SYCE1
SPRNP1

Figure S8. Hierarchical boosting scores and selection tests from PopHuman Browser Phase 3 for *CYP4B1* and *CYP4Z1*. a) F_{ST} comparison of CHB with CEU or YRI show values above 2 sd of the genome-wide mean in *CYP4Z1*. iHS scores in CEU are above 2 sd of the genome-wide mean in both genes, whereas CHB only show significant values of iHS in *CYP4B1*. Some Indian populations (GIH) show significant values of iHS in *CYP4Z1*. b) However, HB scores do not show evidence of positive selection in any of the three continental populations in these genes. Significant scores are highlighted by a red square, gene region is delimitated by a black square.

a)

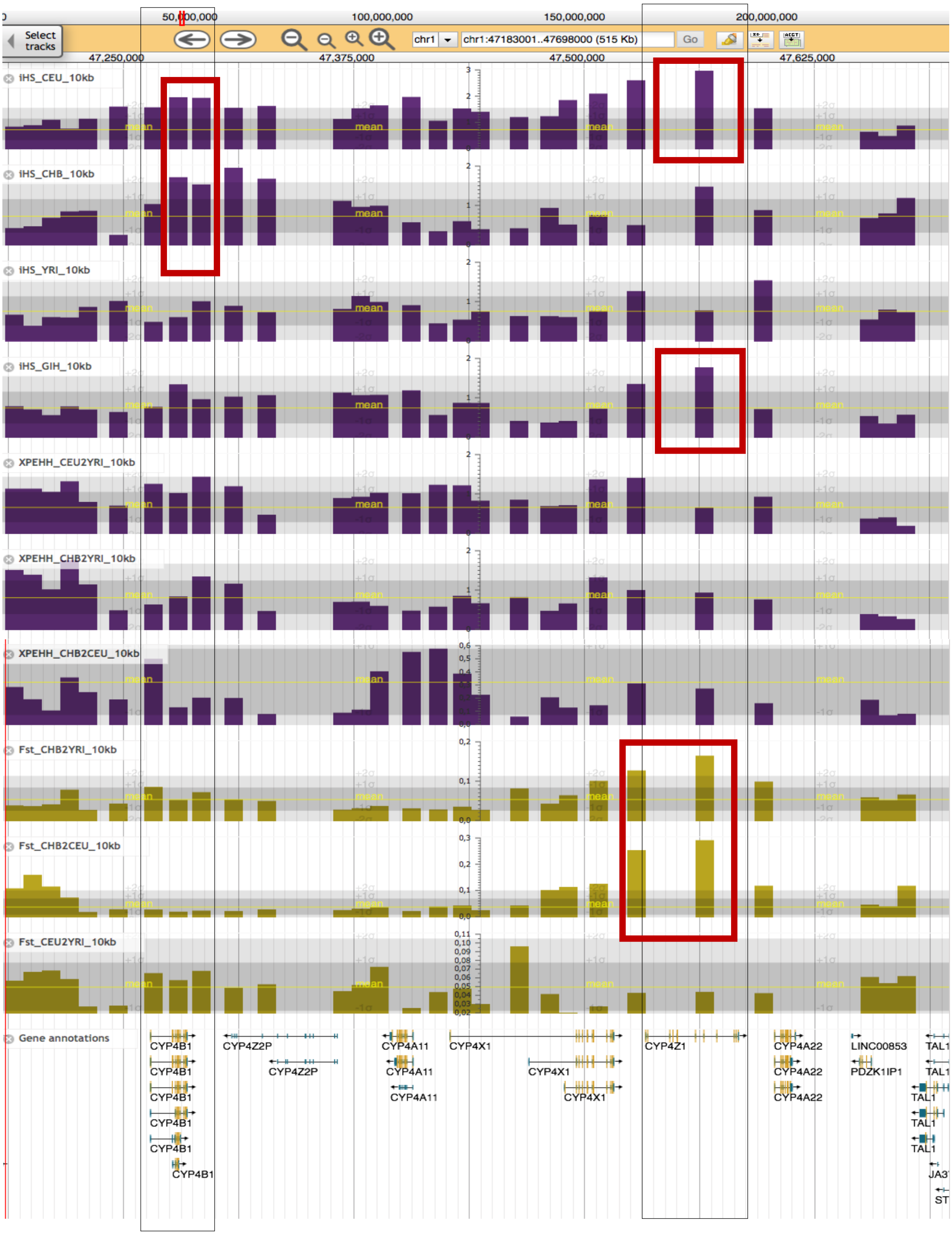

b)

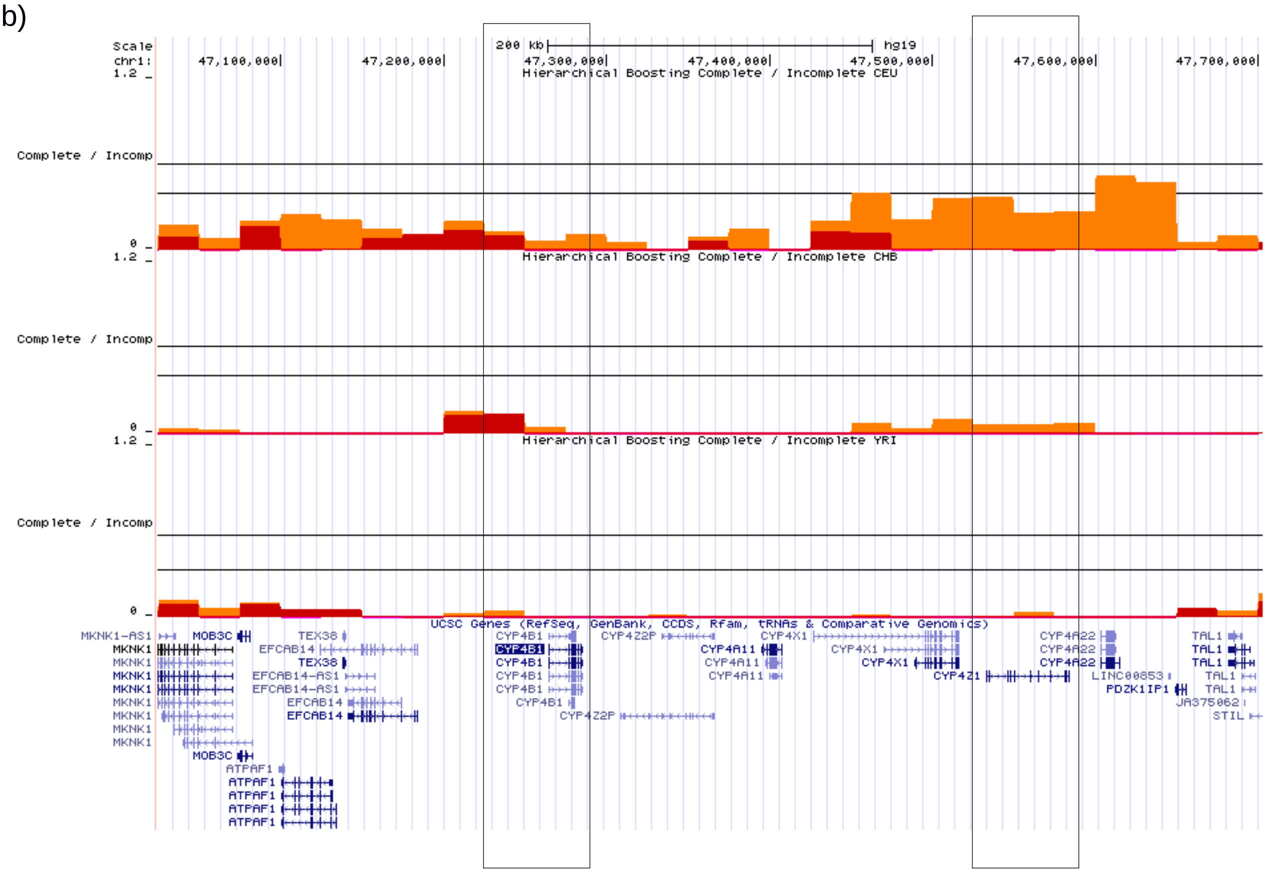
Scale
chr1:
200 kb
hg19
47,100,000
47,200,000
47,300,000
47,400,000
47,500,000
47,600,000
47,700,000
Hierarchical Boosting Complete / Incomplete CEU
Hierarchical Boosting Complete / Incomplete CHB
Hierarchical Boosting Complete / Incomplete YRI
Complete / Incomp
1.2
0
UCSC Genes (RefSeq, GenBank, CCDS, Rfam, tRNAs & Comparative Genomics)
MKNK1-AS1
MKNK1
MOB3C
ATPAF1
TEX38
EFCAB14
EFCAB14-AS1
CYP4B1
CYP4Z2P
CYP4A11
CYP4X1
CYP4Z1
CYP4A22
LINC00853
PDZK1IP1
TAL1
JA375062
STIL

Figure S9. Selection tests from PopHuman Browser Phase 3 for *CYP4F12*. a) iHS tracks showing African populations with scores above 2 sd of the genome-wide mean; b) F_{ST} comparison between the three continental populations. The comparison of CEU with CHB gives scores above 2 sd of the genome-wide mean. There is no HB information for that genomic region. Significant scores are highlighted by a red square, gene region is delimitated by a black square.

a)

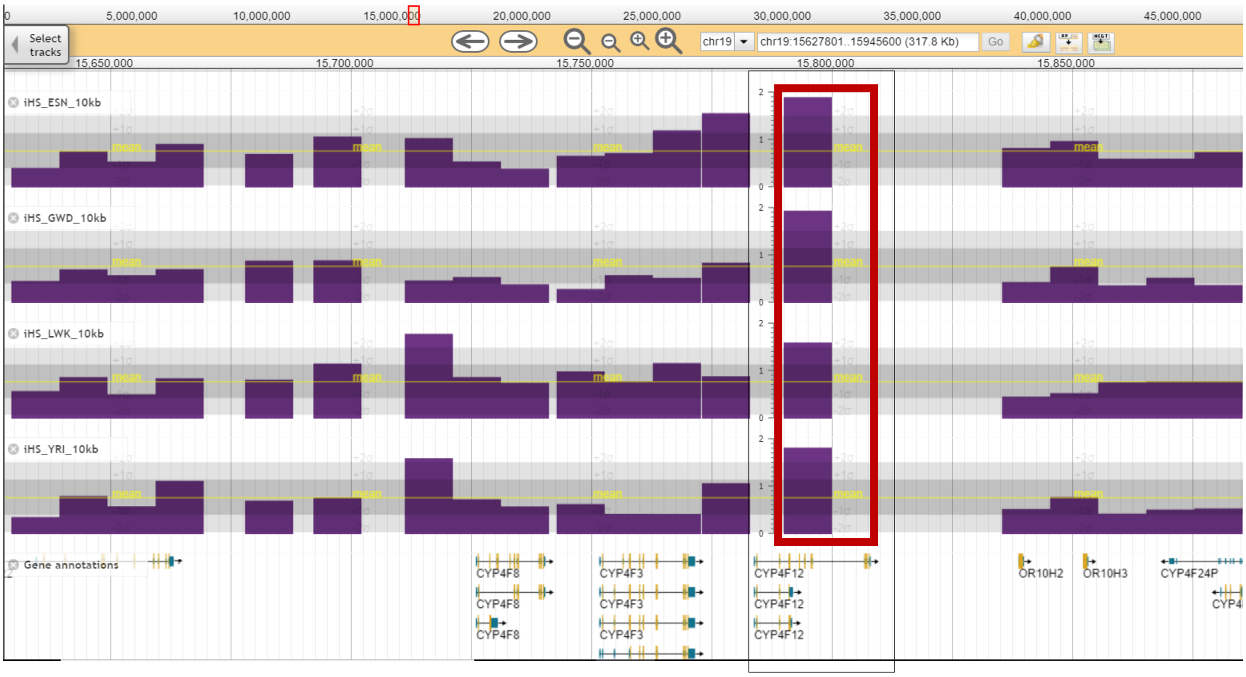

b)

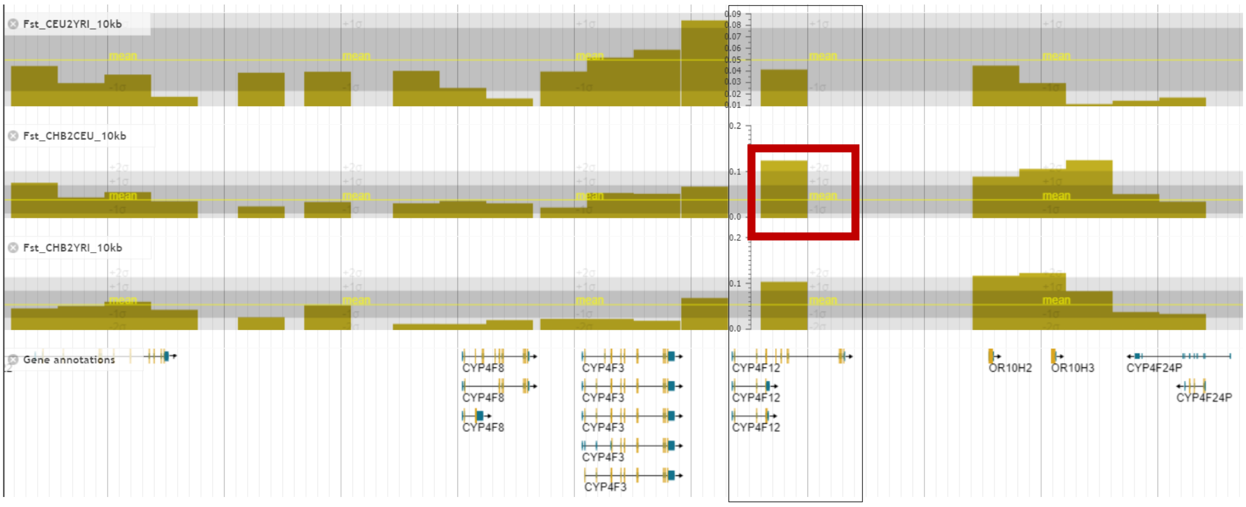

Figure S10. Selection tests from the 1000 Genomes Selection Browser 1.0 and PopHuman Browser Phase 3 in the *CYP3* locus. a) Selection tests from the 1000 Genomes Selection Browser 1.0 showing significant scores of deltaDAF when comparing the three continental populations and XP-CLR when comparing CEU and YRI; b) PopHuman XP-EHH tracks showing scores above 2 sd of the genome-wide mean when comparing YRI with CEU or CHB in *CYP3A7* and *CYP3A4*; c) PopHuman F_{ST} tracks showing scores above 2 sd of the genome-wide mean in *CYP3A4*, *CYP3A7* and *CYP3A43* when comparing YRI with CEU, and in *CYP3A43* when comparing YRI with CHB; d) PopHuman iHS tracks in Asian populations tracks showing scores above 2 sd of the genome-wide mean in *CYP3A5, CYP3A7*, and *CYP3A4*. Significant scores are highlighted by a red square, gene region is delimitated by a black square.

a)

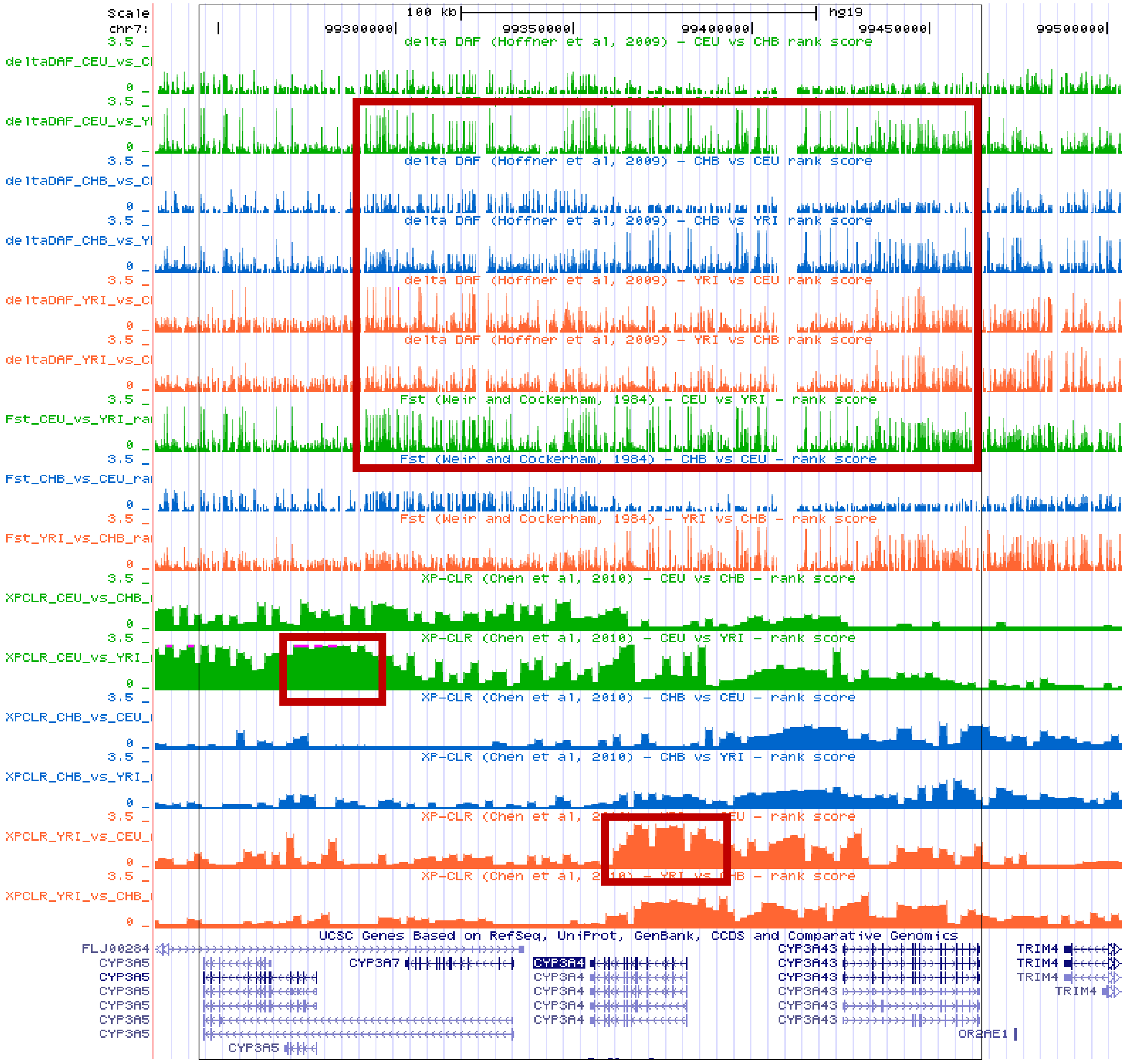

b)

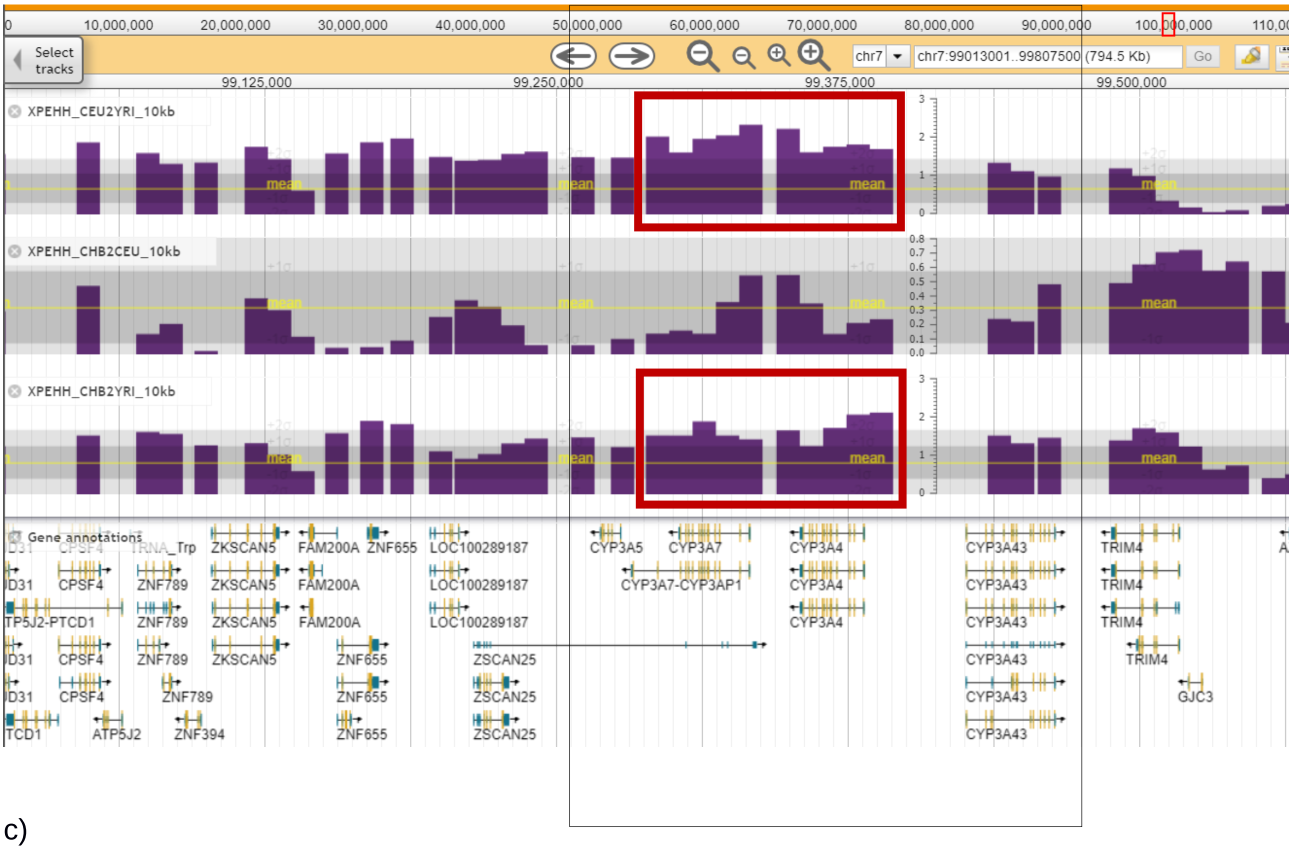

0
10,000,000
20,000,000
30,000,000
40,000,000
50,000,000
60,000,000
70,000,000
80,000,000
90,000,000
100,000,000
Select tracks
chr7
chr7:99013001..99807500 (794.5 Kb)
Go
99,125,000
99,250,000
99,375,000
99,500,000
XPEHH_CEU2YRI_10kb
XPEHH_CHB2CEU_10kb
XPEHH_CHB2YRI_10kb
mean
Gene annotations
CPSF4
ZKSCAN5
FAM200A
ZNF655
LOC100289187
CYP3A5
CYP3A7
CYP3A4
CYP3A43
TRIM4
CYP3A7-CYP3AP1
ZNF789
TP5J2-PTCD1
ZSCAN25
GJC3
ATP5J2
ZNF394

c)

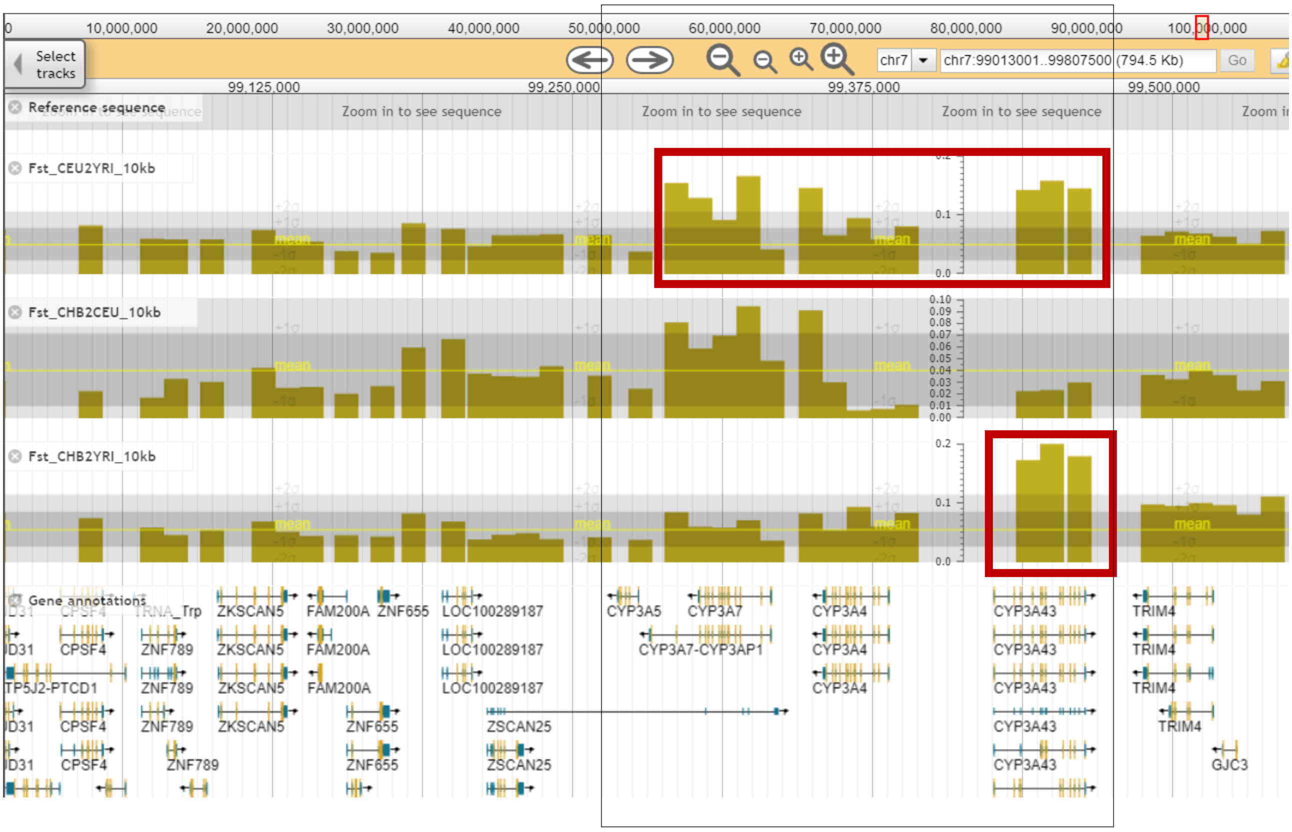

0
10,000,000
20,000,000
30,000,000
40,000,000
50,000,000
60,000,000
70,000,000
80,000,000
90,000,000
100,000,000
Select tracks
chr7
chr7:99013001..99807500 (794.5 Kb)
Go
99,125,000
99,250,000
99,375,000
99,500,000
Reference sequence
Zoom in to see sequence
Fst_CEU2YRI_10kb
Fst_CHB2CEU_10kb
Fst_CHB2YRI_10kb
mean
Gene annotations
CPSF4
ZKSCAN5
FAM200A
ZNF655
LOC100289187
CYP3A5
CYP3A7
CYP3A4
CYP3A43
TRIM4
CYP3A7-CYP3AP1
ZNF789
TP5J2-PTCD1
ZSCAN25
GJC3

d)

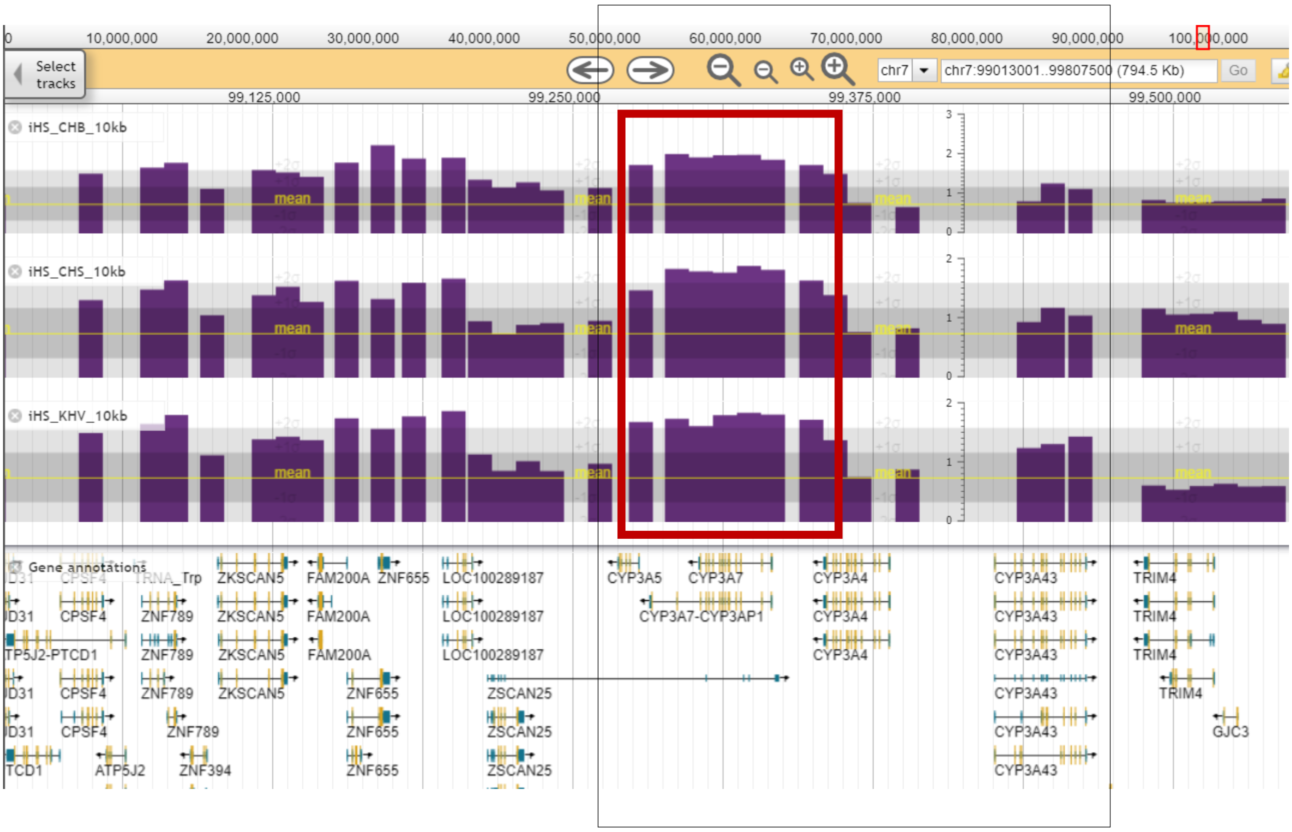

[iHS tracks from PopHuman showing populations from 1000 Genomes Project Phase 3]

Figure S11. Selection tests from PopHuman Browser Phase 3 in the *CYP27A1* gene. a) Selection tests in PopHuman showing XP-EHH tracks with scores above 2 sd of the genome-wide mean comparing YRI with CEU or CHB; b) PopHuman F_{ST} tracks with scores above 2 sd of the genome-wide mean comparing CHB with CEU or YRI. Significant scores are highlighted by a red square, gene region is delimitated by a black square.

a)

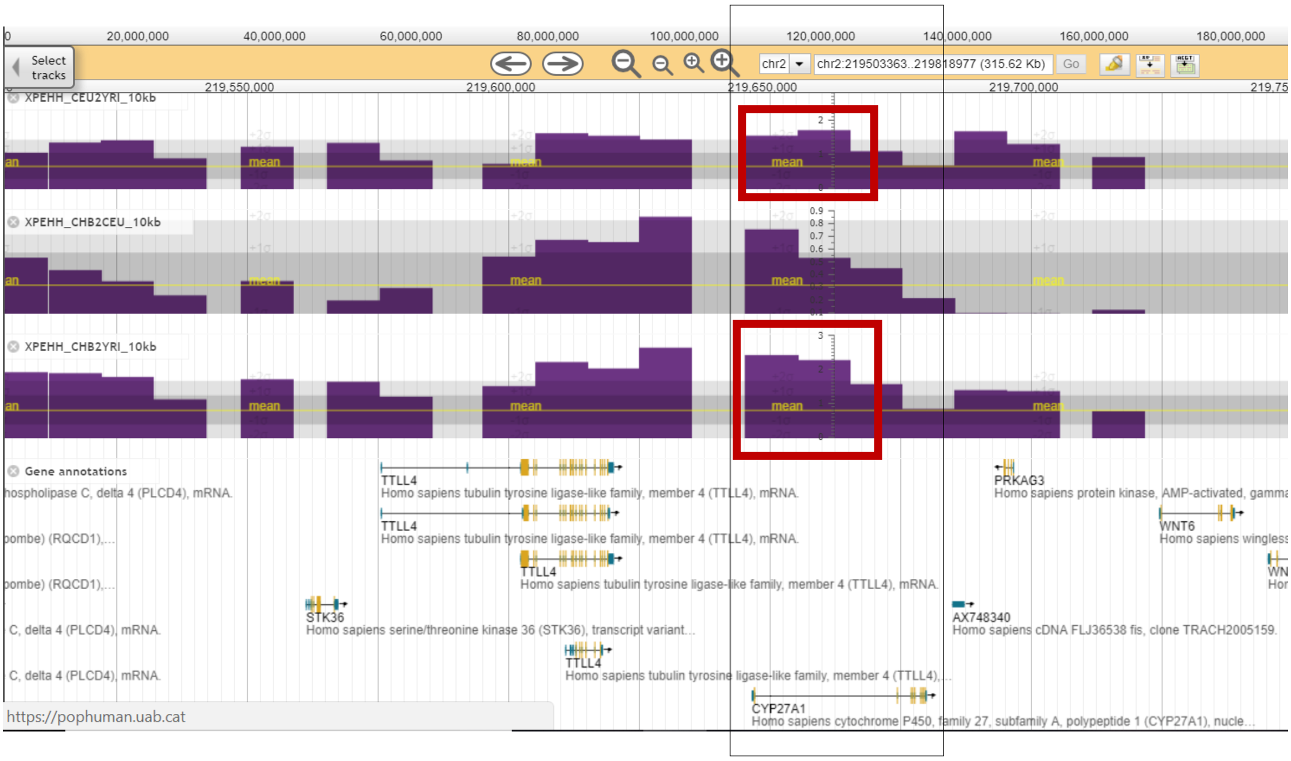

b)

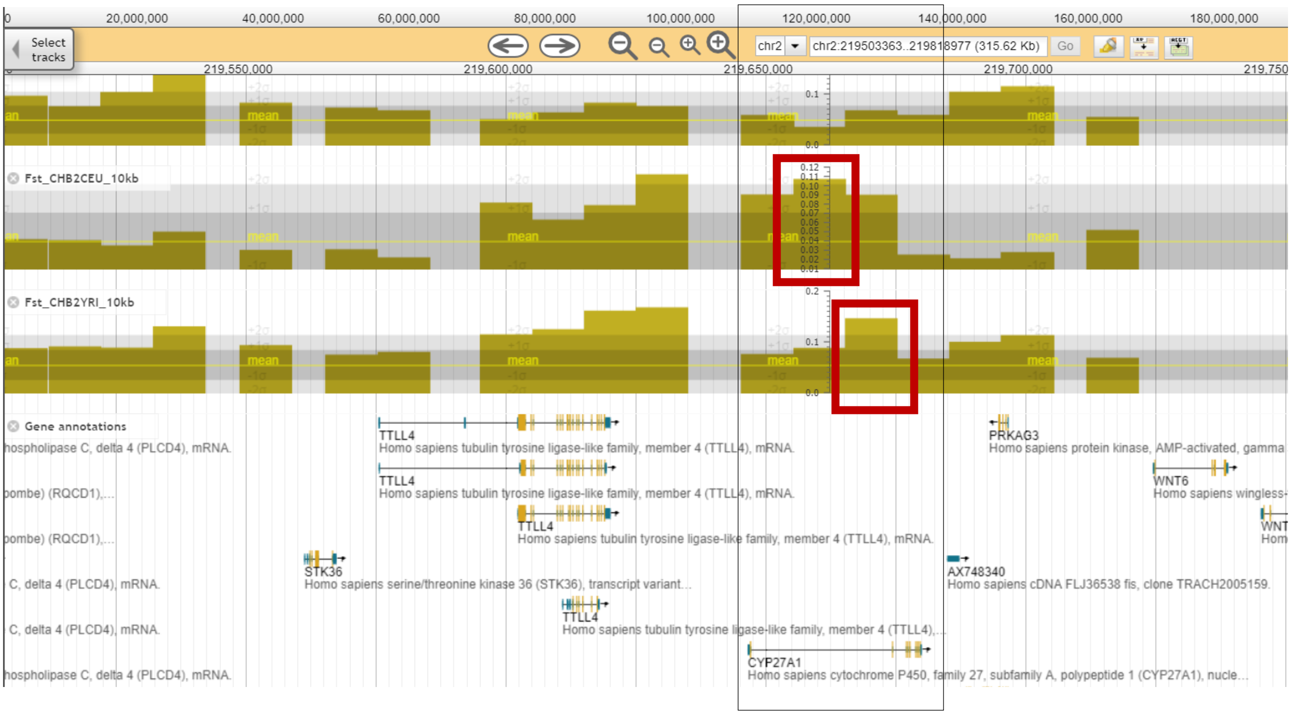

References

1. Pybus M, Dall'Olio GM, Luisi P, Uzkudun M, Carreño-Torres A, Pavlidis P, et al. 1000 Genomes Selection Browser 1.0: a genome browser dedicated to signatures of natural selection in modern humans. Nucleic Acids Res. 2014;42:D903–9.
2. Weir BS, Cockerham CC. Estimating F-Statistics for the Analysis of Population Structure. Evolution (N Y). 1984;38:1358.
3. Voight BF, Kudaravalli S, Wen X, Pritchard JK. A Map of Recent Positive Selection in the Human Genome. PLoS Biol. 2006;4:e72.
4. Tajima F. Statistical method for testing the neutral mutation hypothesis by DNA polymorphism. Genetics. 1989;123:585–95.
5. Sabeti PC, Varilly P, Fry B, Lohmueller J, Hostetter E, Cotsapas C, et al. Genome-wide detection and characterization of positive selection in human populations. Nature. 2007;449:913–8.
6. Chen H, Patterson N, Reich D. Population differentiation as a test for selective sweeps. Genome Res. 2010;20:393–402.
7. Pybus M, Luisi P, Dall'Olio GM, Uzkudun M, Laayouni H, Bertranpetit J, et al. Hierarchical boosting: a machine-learning framework to detect and classify hard selective sweeps in human populations. Bioinformatics. 2015;31:btv493.
8. Casillas S, Mulet R, Villegas-Mirón P, Hervas S, Sanz E, Velasco D, et al. PopHuman: the human population genomics browser. Nucleic Acids Res. 2018;46:D1003–10.
9. Hudson RR, Slatkin M, Maddison WP. Estimation of levels of gene flow from DNA sequence data. Genetics. 1992;132:583–9.
